# The spatiotemporal matching pattern of Ezrin/Periaxin involved in myoblast differentiation and fusion and Charcot-Marie-Tooth disease-associated muscle atrophy

**DOI:** 10.1186/s12967-023-04016-7

**Published:** 2023-03-04

**Authors:** Ruo-nan Zhang, Xin Bao, Yun Liu, Yan Wang, Xing-Yuan Li, Ge Tan, Magdaleena Naemi Mbadhi, Wei Xu, Qian Yang, Lu-yuan Yao, Long Chen, Xiao-ying Zhao, Chang-qing Hu, Jing-xuan Zhang, Hong-tao Zheng, Yan Wu, Shan Li, Shao-juan Chen, Shi-you Chen, Jing Lv, Liu-liu Shi, Jun-ming Tang

**Affiliations:** 1 Faculty of Basic Medical Sciences, Postgraduate Union Training Basement of Jin Zhou Medical University, Hubei University of Medicine, Shiyan, 442000 Hubei People’s Republic of China; 2 Institute of Anesthesiology, Department of Anesthesiology, Hubei University of Medicine, Shiyan, 442000 Hubei People’s Republic of China; 3 Department of Physiology, Hubei Key Laboratory of Embryonic Stem Cell Research,Faculty of Basic Medical Sciences, Hubei University of Medicine, Shiyan, 442000 Hubei People’s Republic of China; 4 Emergency Comprehensive Department, Shiyan Maternal and Child Health Hospital, Hubei University of Medicine, Shiyan, 442000 Hubei People’s Republic of China; 5 Department of Physiology, Faculty of Basic Medical Sciences, Zunyi Medical University, Zunyi, 563006 Guizhou People’s Republic of China; 6 Experimental Medical Center, Dongfeng Hospital, Hubei University of Medicine, Shiyan, China; 7 Department of Biochemistry, Faculty of Basic Medical Sciences, Hubei University of Medicine, Shiyan, 442000 Hubei People’s Republic of China; 8 Department of Stomatology, Taihe Hospital, Hubei University of Medicine, Shiyan, 442000 Hubei People’s Republic of China; 9 Department of Surgery, University of Missouri, Columbia, USA

**Keywords:** Ezrin, L-periaxin, Myoblast, Myotube, PKA, NFAT, MEF2c

## Abstract

**Background:**

Clinically, Charcot-Marie-Tooth disease (CMT)-associated muscle atrophy still lacks effective treatment. Deletion and mutation of L-periaxin can be involved in CMT type 4F (CMT4F) by destroying the myelin sheath form, which may be related to the inhibitory role of Ezrin in the self-association of L-periaxin. However, it is still unknown whether L-periaxin and Ezrin are independently or interactively involved in the process of muscle atrophy by affecting the function of muscle satellite cells.

**Method:**

A gastrocnemius muscle atrophy model was prepared to mimic CMT4F and its associated muscle atrophy by mechanical clamping of the peroneal nerve. Differentiating C2C12 myoblast cells were treated with adenovirus-mediated overexpression or knockdown of Ezrin. Then, overexpression of L-periaxin and NFATc1/c2 or knockdown of L-periaxin and NFATc3/c4 mediated by adenovirus vectors were used to confirm their role in Ezrin-mediated myoblast differentiation, myotube formation and gastrocnemius muscle repair in a peroneal nerve injury model. RNA-seq, real-time PCR, immunofluorescence staining and Western blot were used in the above observation.

**Results:**

For the first time, instantaneous L-periaxin expression was highest on the 6th day, while Ezrin expression peaked on the 4th day during myoblast differentiation/fusion in vitro. In vivo transduction of adenovirus vectors carrying Ezrin, but not Periaxin, into the gastrocnemius muscle in a peroneal nerve injury model increased the numbers of muscle myosin heavy chain (MyHC) I and II type myofibers, reducing muscle atrophy and fibrosis. Local muscle injection of overexpressed Ezrin combined with incubation of knockdown L-periaxin within the injured peroneal nerve or injection of knockdown L-periaxin into peroneal nerve-injured gastrocnemius muscle not only increased the number of muscle fibers but also recovered their size to a relatively normal level in vivo*.* Overexpression of Ezrin promoted myoblast differentiation/fusion, inducing increased MyHC-I^+^ and MyHC-II + muscle fiber specialization, and the specific effects could be enhanced by the addition of adenovirus vectors for knockdown of L-periaxin by shRNA. Overexpression of L-periaxin did not alter the inhibitory effects on myoblast differentiation and fusion mediated by knockdown of Ezrin by shRNA in vitro but decreased myotube length and size. Mechanistically, overexpressing Ezrin did not alter protein kinase A gamma catalytic subunit (PKA-γ cat), protein kinase A I alpha regulatory subunit (PKA reg Iα) or PKA reg Iβ levels but increased PKA-α cat and PKA reg II α levels, leading to a decreased ratio of PKA reg I/II. The PKA inhibitor H-89 remarkably abolished the effects of overexpressing-Ezrin on increased myoblast differentiation/fusion. In contrast, knockdown of Ezrin by shRNA significantly delayed myoblast differentiation/fusion accompanied by an increased PKA reg I/II ratio, and the inhibitory effects could be eliminated by the PKA reg activator N6-Bz-cAMP. Meanwhile, overexpressing Ezrin enhanced type I muscle fiber specialization, accompanied by an increase in NFATc2/c3 levels and a decrease in NFATc1 levels. Furthermore, overexpressing NFATc2 or knocking down NFATc3 reversed the inhibitory effects of Ezrin knockdown on myoblast differentiation/fusion.

**Conclusions:**

The spatiotemporal pattern of Ezrin/Periaxin expression was involved in the control of myoblast differentiation/fusion, myotube length and size, and myofiber specialization, which was related to the activated PKA-NFAT-MEF2C signaling pathway, providing a novel L-Periaxin/Ezrin joint strategy for the treatment of muscle atrophy induced by nerve injury, especially in CMT4F.

**Supplementary Information:**

The online version contains supplementary material available at 10.1186/s12967-023-04016-7.

## Introduction

Charcot-Marie-Tooth disease (CMT) is mainly attributed to hereditary motor sensory neuropathological changes characterized by loss or reduction of the myelin sheath [1–4]. The main clinical manifestation of CMT is progressive weakness and atrophy of the distal limb muscles with sensory disturbance [1, 4]. Clinically, there is no good treatment for CMT-associated muscle atrophy, and it is urgent to explore new treatment strategies because muscle atrophy not only affects quality of life but also brings heavy social and economic burdens [3].

As one of the CMT subtypes, the occurrence and development of Charcot-Marie-Tooth disease type 4F (CMT4F) can be mainly attributed to the deletion and mutation of the periaxin gene located at 19q13, which causes nerve demyelination and its associated muscle atrophy at the distal end of the lower limb, especially the gastrocnemius muscle [4]. The periaxin gene encodes two periaxin isoform proteins, the full-length segment (L-periaxin) and short segment (S-periaxin) [1]. In the process of forming the myelin sheath, the periaxin, especially the L-periaxin, participates in the interaction of membrane proteins, which is necessary to maintain the maturation of the myelin sheath [4]. Furthermore, the Ezrin protein encoded by the villin2 gene maintains the stability of the myelin sheath by blocking the self-association of L-periaxin [5]. However, in addition to the pathological changes in the nerve itself, it is still unclear whether demyelinating-associated CMT4F is related to the regeneration and repair ability of the skeletal muscle it innervates.

Indeed, muscle satellite cells play a particularly critical role in physiological self-renewal and pathological damage repair in skeletal muscle [6, 7]. Recently, our group and other scholars found that activated PKA and alterations in both PKA regulatory subunit I (PKA RI) and PKA RII are involved in myoblast differentiation/fusion and myotube formation [8, 9]. Meanwhile, Ezrin can anchor cAMP-dependent protein kinases, resulting in the activation of protein kinase A (PKA) and phosphorylation of the Na^+^/H^+^ exchanger (NHE3) [10, 11]. Although it is still unknown whether the change in Ezrin in skeletal muscle H^+^ secretion is the same as its classical role in regulating gastric acid secretion, the proper increase in H^+^ secretion marked by the appropriate reduction in pH is beneficial to the regeneration and repair of skeletal muscle [12, 13]. Furthermore, Ezrin can adjust the self-association of L-periaxin [5]. These results pushed us to speculate that Ezrin and/or L-periaxin can be involved in the regulation of muscle satellite cells and the regeneration and repair of skeletal muscle, especially CMT4F-related muscle atrophy.

Of interest, NFATs (nuclear factor of activated T cells) activated by PKA contribute to myoblast differentiation/fusion and myotube formation [14–16]. In this study, for the first time, the matching pattern of Ezrin/Periaxin expression regulated myoblast differentiation/fusion, myotube length and size, and myofiber specialization, and the combination of injected adenovirus vector carrying Ezrin (Ad-Ezrin) into the gastrocnemius muscle (GA) with injection of Ad-shPeriaxin into the GA or incubation of Ad-Periaxin within the injured peroneal nerve increased the number of MyHC-I/II myofibers, resulting in the recovery of GA atrophy in a peroneal nerve injury model, providing a novel therapeutic strategy for muscle atrophy, especially in CMT4F.

## Method

### Animals, in vivo transfection and peroneal nerve injury model preparation

Animal studies were performed according to the Guide for the Care and Use of Laboratory Animals published by the US National Institutes of Health and China. The Experimental Animal Centre of Hubei Medical University provided C57BL/6 mice (male, 3–5 months) that met the criteria. The Institutional Animal Care and Use Committee of Hubei Medical University approved the animal protocols (Cat: 2019–111).

For the transduction of adult muscles, C57BL/6 male mice were anesthetized by using an isoflurane vaporizer maintained at 2% isoflurane and 1 L/m oxygen. Gastrocnemius and soleus (SL) muscles were exposed and injected with Ad-Ezrin (1 × 10^10^ pfu, two points, 50 μm/each) [15]. Muscles were removed 7 days after transfection, frozen in isopentane cooled in liquid nitrogen, and stored at − 80 °C.

Mutation and deletion of L-periaxin was associated with Charcot-Marie-Tooth (CMT) characterized by progressive muscle weakness and atrophy of distal extremities with sensory impairment through destroying the myelin sheath formed by Schwann cells. Interestingly, Ezrin inhibits the self-association of L-periaxin and participates in myelin sheath maintenance [4, 5]. To confirm whether L-periaxin/Ezrin independence and interaction participate in CMT and muscular atrophy, a peroneal nerve injury model was prepared. Briefly, C57BL/6 male mice were anesthetized by using an isoflurane vaporizer maintained at 2% isoflurane and 1 L/m oxygen. Peroneal nerves were exposed and clamped for 15 min; subsequently, the gastrocnemius muscle (GA) was injected with Ad-Ezrin, Ad-Periaxin or Ad-shPeriaxin alone (1 × 10^10^ pfu, three points, 50 μm/each), and combined treatment with Ad-Ezrin (1 × 10^10^ pfu, three points, 50 μm/each) injection into the GA with Ad-shPeriaxin injection into the GA or Ad-Periaxin incubation within the injured peroneal nerves was incubated with Ad-Periaxin (1 × 10^10^ pfu, 50 μm/each) [15]. The sham and PNI groups within the peroneal nerves and GA were treated with equal amounts of normal saline. Muscles were removed 14 days after transfection, frozen in isopentane cooled in liquid nitrogen, and stored at − 80 °C. The myofiber types were measured through double fluorescence immunostaining of MyHC-I (NOQ, ab234431) and MyHC-II (My32, ab51263). Masson and hematoxylin-eosin staining were performed according to the manufacturer’s instructions. The successful establishment of the peroneal nerve injury model is shown in Additional file 1: Figure S1.

### C2C12 myoblast culture and differentiation induction

C2C12 myoblasts (Cat: SCSP-505, purchased from the Cell Resource Center of Shanghai Academy of Life Sciences, Chinese Academy of Sciences) were inoculated in 75-cm^2^ culture dishes and cultured with proliferation medium (PM) containing high-glucose DMEM (Gibco, USA, HG-DMEM) supplemented with 10% FBS (Gibco, USA) at 37 °C and 5% CO_2_. When the confluence of the cells reached 75%, the PM was replaced with differentiation medium (DM) containing HG-DMEM supplemented with 2% horse serum (HS, Sigma, USA) to induce C2C12 myoblast cell differentiation. Traits of myotube formation from myoblast differentiation were observed daily under a microscope [14].

### Adenoviral vector preparation and in vitro transfection

Ezrin-, L-periaxin-, and NFATc1/c2-overexpressing adenoviral vectors were prepared as previously described [15]. The gene accession numbers of overexpressing-Ezrin, L-periaxin, and NFATc1/c2 are NM_172390 and NM_173091, respectively. The adenoviral vectors carrying short hairpin RNA (shRNA) for knockdown of Ezrin, L-periaxin and NFATc3/c4 were prepared as previously described (Hicks et al., 2014). These overexpression adenoviral vectors containing Ad-NFATc1, Ad-NFATc2, Ad-shNFATc3 and Ad-shNFATc4 were obtained from Vigenebio. To confirm the role of L-periaxin in myoblasts, Ad-Null, Ad-Periaxin, or Ad-shPeriaxin (1 × 10^9^ pfu) was added to the corresponding culture dishes one day before Ad-Ezrin or Ad-shEzrin was added. To confirm the role of NFATc3 or NFAtc4 in myoblasts, Ad-Null, Ad-shNFATc3, or Ad-shNFATc4 (1 × 10^9^ pfu) was added to the corresponding culture dish one day before Ad-Ezrin or Ad-shEzrin was added. Then, the proliferation medium was replaced with differentiation medium for further observation. The successful knockdown and overexpression of exogenous genes was measured by detecting the His-tag, Ezrin and L-periaxin (Additional file 1: Figure S2–S3).

### Immunofluorescence staining

C2C12 myoblast differentiation was determined by immunofluorescence staining. Primary monoclonal and polyclonal antibodies against MEF2C (#5030 s, 1:200, CST), MyoG (sc-12732, 1:150, Santa Cruz), MyHC (sc-20641, 1:150, Santa Cruz), MyHC-I (NOQ, 1:200, ab234431) and MyHC-II (My32, 1:200, ab51263) were added to each well in every group and incubated for 12 h at 4 °C. The cells were washed with PBS 3 times for 15 min and incubated with appropriate fluorescent dye-labeled secondary antibodies (Jackson Lab, 1:500, USA) at 25 °C for 2 h. The nuclei were stained with DAPI (Molecular Probes). The images for each group were photographed under a Nikon 80i fluorescence microscope [16].

### Myoblast differentiation

After myoblasts were treated with differentiation medium (DM) containing HG-DMEM supplemented with 2% horse serum (HS, Sigma, USA) for the indicated time, the differentiated myoblasts were stained for MyoG or MEF2C using the primary polyclonal antibody MyoG (sc-12732, 1:150, Santa Cruz) or MEF2C (5030S, 1:400, CST) and the appropriate TRITC-labeled secondary antibody (Jackson Lab, 1:500, USA). The nuclei were stained with DAPI. C2C12 myoblasts with only 1–2 nuclei within a cellular structure were evaluated with MyoG or MEF2C staining. MyoG + or MEF2C + cells were defined as differentiated cells that did not fuse to form myotubes. Myoblasts with 3 or more nuclei in the structure of a cell were defined as myotubes. The number of double-positive nuclei in a high-power field (HPF, 50 μm) was analyzed after double staining with MyoG/DAPI or MEF2C/DAPI. Two individuals who were blinded to the results evaluated the images using ImageJ (Java) software (National Institutes of Health, USA).

### Myoblast fusion and myotube morphology

The differentiated myoblasts were stained for MyHC with the primary polyclonal antibody MyHC (rabbit anti-mouse antibody, sc-20641, 1:150, Santa Cruz) and the appropriate TRITC or FITC-labeled secondary antibody (Jackson Lab, 1:500, USA). C2C12 myoblasts with only 1–2 nuclei within a cellular structure were evaluated by MyHC staining, indicating that MyHC + cells were defined as differentiated cells without mutual fusion to myotubes. Myoblasts with 3 or more nuclei in the structure of a cell were defined as myotubes. The nuclei were stained with DAPI.

To analyze myotube size, we divided the cells into 2 groups, including short myotubes with 3 ~ 5 myoblast fusions and long myotubes with more than 5 myoblast fusions. Morphology was assessed by myotube length, area (grouped less than 200 μm and more than 200 μm), and the number of myotubes (grouped 3 ~ 5 nuclei or more than 5 myoblast fusion nuclei) under high-power magnification [15, 16]. To describe the traits of myotubes with more than 5 myoblast fusions, the myotube length and size were assessed by an alteration index as defined by the actual length of the control group divided by the actual length of the treatment group [15, 16]. Three independent experiments were carried out, three repetitions each time, and five fields of vision were randomly selected for each repetition. Two individuals who were blinded to the results evaluated the images using ImageJ (Java) software (National Institutes of Health, USA).

### RNA sequencing analysis

RNA-seq was carried out in murine C2C12 myoblasts treated with differentiation medium containing Ad-Null, Ad-Ezrin or Ad-shEzrin (1 × 10^9^ pfu) for 6 days. Library preparations were sequenced on an MGISEQ-T7 platform at OE Biotech Co., Ltd. (Shanghai, China), and approximate 150 bp paired-end reads were generated when quality inspection was finished. After removing the adaptor and low-quality sequence reads from the data sets by using the software Trimmomatic. These clean reads were then mapped to the peach reference genome sequence. Using htseq-count software, the number of reads were obtained on the protein-coding gene of each sample. Subsequently, cufflinks software was used to calculate the fragments per kilobase of exon per million mapped fragments (FPKM) value of the protein-coding gene expression. And then, using the R package DEseq (2012), differential gene expression analysis was done through setting the threshold (P value < 0.05 and fold change > 2 or fold change < 0.5) to determine significantly differential expression. Finally, hierarchical cluster analysis of the differentially expressed genes (DEGs) was executed to show the pattern of genes expressions among the three groups and samples. Furthermore, R based on the hypergeometric distribution was used to analyzed Kyoto Encyclopedia of Genes and Genomes (KEGG) pathway enrichment and Gene Ontology (GO) enrichment of DEGs [17].

### Quantitative RT‒PCR

Total RNA from C2C12 myoblasts was obtained using TRIzol (Invitrogen, Life Technologies) and transcribed into cDNA using the SuperScript II cDNA kit (Invitrogen, Life Technologies). Quantitative PCR was carried out using SYBR green PCR master mix (Thermo Fisher Scientific, Applied Biosystems, CN) in a real-time PCR system (RotorGene 6000, Qiagen, Germany). The transcript levels of the gene of interest in each group were normalized to the GAPDH levels [18]. The primers used are listed in Table 1.

**Table1:** The sequences of primers of qPCR

Gene	Forward	Reverse
MyoG	5′-GAGACATCCCCCTATTTCTACCA-3′	5′-GCTCAGTCCGCTCATAGCC-3′
MyoD1	5′-CCACTCCGGGACATAGACTTG-3′	5′-AAAAGCGCAGGTCTGGTGAG-3′
MyHC1	5′-CAAGCAGCAGTTGGATGAGCGACT-3′	5′-TCCTCCAGCTCCTCGATGCGT-3′
MyHC2a	5′-AGAGGACGACTGCAGACCGAAT-3′	5′-GAGTGAATGCTTGCTTCCCCCTTG-3′
MyHC2b	5′-ACGCTTGCACACAGAGTCAG-3′	5′-CTTGGACTCTTCCTCTAGCTGCC-3′
MyHC2x	5′-ACCAAGGAGGAGGAACAGCAGC-3′	5′-GAATGCCTGTTTGCCCCTGGAG-3′
GAPDH	5′-ATGACTCCACTCACGGCAAA-3′	5′-ATGATGACCCTTTTGGCTCC-3′

qPCRs were performed to identified satellite cell differentiation and muscle fibers traits by using the specific primers of satellite cell differentiation markers including MyoD and MyoG, type I muscle fiber makers like MyHC1, and type II muscle fiber makers such as MyHC2a, MyHC2b, and MyHC2X

### Western blot

C2C12 myoblasts were homogenized on ice in 0.1% Tween-20 homogenization buffer containing protease inhibitors. Nuclear and cytosolic proteins were separated and collected using NE-PER Nuclear and Cytoplasmic Extraction Reagents according to the manufacturer’s instructions (78,835, Thermo Fisher Scientific, USA). Twenty micrograms of protein in each well was separated by 7 or 10% SDS‒PAGE and transferred onto PVDF membranes (Millipore). After blocking with 5% nonfat milk, the membranes were incubated with primary antibodies against α-tubulin (T9026, 1:5000, Sigma), histone H3 (ab6002, 1:500, ABCAM), NFATc1 (ab2796, 1:500, Abcam), NFATc2 (ab2722, 1:500, Abcam), NFATc3 (ab83832, 1:500, Abcam), NFATc4 (SAB4501982, 1:1000, Sigma) and MyHC (sc-20641, sc-376157, 1:500, Santa Cruz) overnight at 4 °C. Thereafter, the blots were incubated with corresponding horseradish peroxidase (HRP)-conjugated secondary antibodies (anti-rabbit IgG, anti-goat IgG, 1:10,000; Santa Cruz) for 90 min. Protein expression was detected by the enhanced chemiluminescence method, and ImageJ software was used for gray value analysis [19].

### Statistical analysis

Data from quantitative and semiquantitative analyses are presented as the mean ± SD. Paired or unpaired Student's t test determined statistical significance between the two groups. One-way ANOVA was used to compare the results for more than two experimental groups to specify the differences between groups. *P* < 0.05 was considered meaningful.

## Results

### Combined gene therapy with Ezrin and L-periaxin repaired PNI-associated muscle atrophy

To explore whether L-periaxin/Ezrin’s independence and interaction participate in CMT4F and its muscle atrophy, a peroneal nerve injury (PNI) model was first prepared to partially mimic CMT4F-associated muscle atrophy, showing the traits of gastrocnemius muscle (GA) atrophy (Additional file 1: Figure. S1), indicating that PNI was successfully established. Then, to further mimic CMT4F-associated muscle atrophy, the local injection of Ad-shPeriaxin into PNI-injured GA showed that the numbers of MyHC-I- and MyHC-II-positive fibers were reduced, compared with those in the PNI model group. These results suggested that the PNI model could partially successfully simulate the pathological changes of CMT4F.

**Fig. 1:**
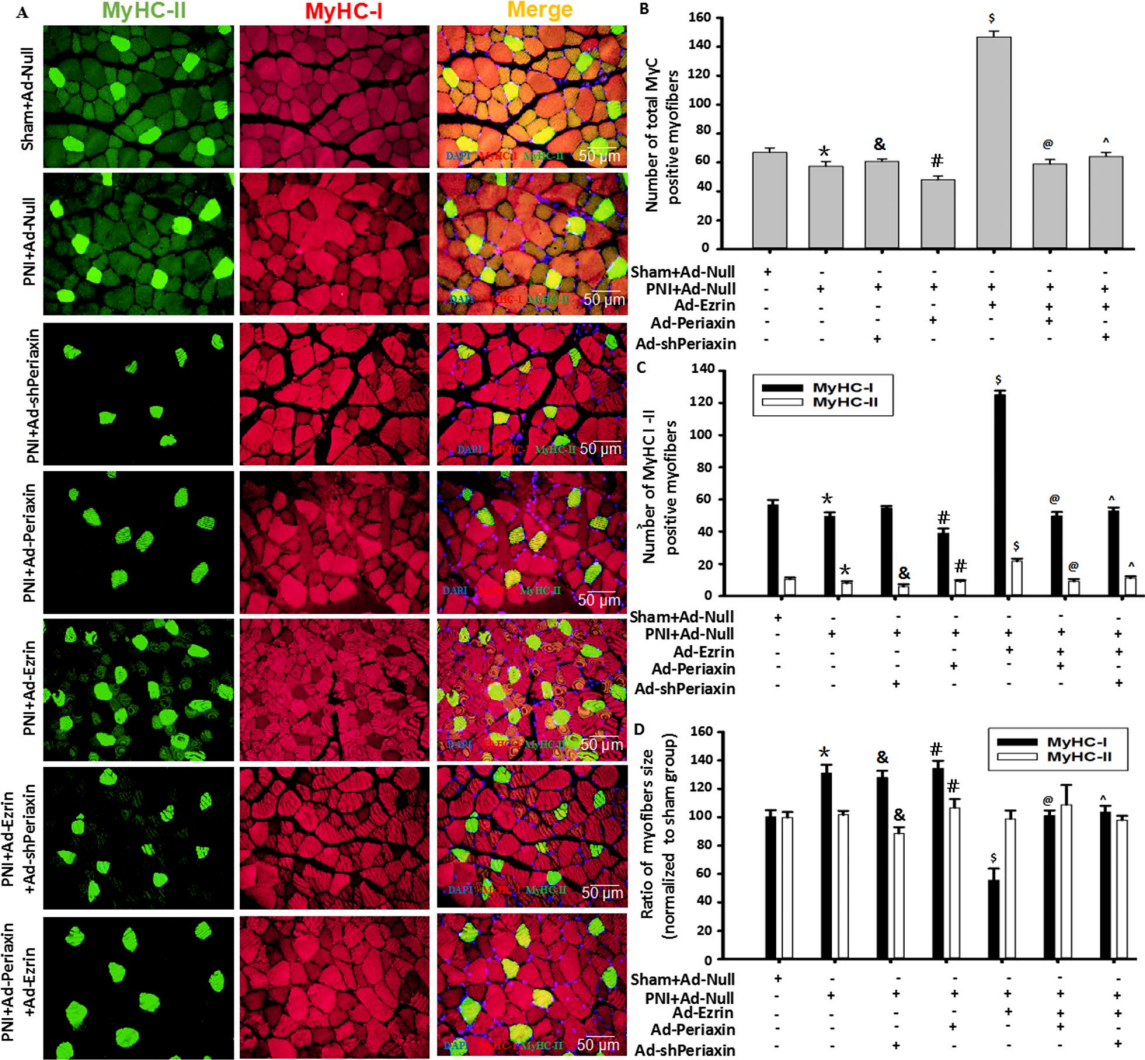
Combined gene therapy with Ezrin and L-periaxin repaired PNI-associated muscle atrophy. **A** Typical image of MyHC-I (NOQ) or MyHC-II (MY32) staining in the gastrocnemius muscle in the peroneal nerve injury (PNI) model. Red fluorescence indicates MyHC-I; green fluorescence indicates MyHC-II; DAPI indicates the nucleus. **B** Quantitative assay for total MyHC-positive myofibers in the above images. Total MyHC + myofiber numbers = MyHC-I + MyHC-II myofiber numbers. **C** Quantitative assay for MyHC-I- or MyHC-II-positive myofibers in the above images. **D** The ratio of myofiber size in MyHC-I- or MyHC-II-positive myofibers in the PNI and treatment groups was normalized to that in the sham group. n = 6, *P < *0.05* vs. Sham + Ad-Null group; ^*#*^*P* < *0.05* vs. PNI + Ad-Null group; ^*&*^*P* < *0.05* vs. PNI + Ad-Null group; ^*$*^*P* < *0.05* vs.PNI + Ad-Null group; ^*@*^*P* < *0.05* vs. Ad-Ezrin + PNI group; *^P* < *0.05* vs. Ad-Ezrin + PNI group

To confirm whether L-periaxin could repair CMT4F-associated muscle atrophy in the PNI model, Ad-periaxin was locally injected into the injured GA, which was not as expected. The in vivo* results* showed that overexpression of L-periaxin (Ad-Periaxin) did not increase the total number of muscle fibers in the PNI model (Fig. 1A–D), indicating that Ad-Periaxin did not reverse PNI-induced GA atrophy. Meanwhile, Ad-periaxin did not obviously alter the numbers of MyHC-I- and MyHC-II-positive fibers (Fig. 1A–D), indicating that gene therapy targeting L-periaxin in skeletal muscle alone could not be an effective treatment strategy for CMT4F-associated muscle atrophy.

Unlike L-periaxin, the local injection of Ad-Ezrin into the GA markedly increased not only the total numbers of muscle fibers in the PNI model but also the numbers of MyHC-I- and MyHC-II-positive fibers, indicating that Ezrin could strongly repair GA atrophy. Of interest, Ad-shPeriaxin within the GA partially canceled the enhanced effect of Ad-Ezrin on increasing MyHC-I- and MyHC-II-positive fiber numbers, especially in MyHC-I fibers. However, Ad-shPeriaxin recovered the overly fine MyHC-I-positive fibers mediated by Ad-Ezrin to a relatively normal size in the sham group in vivo (Fig. 1A–D), implying that the execution of the role of Ezrin could depend at least in part on the absence of L-periaxin within the GA. These results suggested that gene therapy targeting Ezrin in skeletal muscle could be a potential candidate treatment strategy for CMT4F-associated muscle atrophy characterized by mutation or loss of L-periaxin.

To confirm the synergistic repair of the neuroskeletal muscle effects of periaxin and Ezrin in CMT4F cells, local incubation of Ad-Periaxin into the injured PN and local injection of Ad-Ezrin into the GA were simultaneously performed. We found that local application of Ad-Periaxin within the PN and Ad-Ezrin within the GA significantly restored MyHC-I-positive myofibers to a relatively normal size in vivo compared with the Ad-Ezrin alone group (Fig. 1A–D). Furthermore, the above neuromuscular synergistic treatment effect was better than that of the combination of Ad-shPeriaxin and Ad-Ezrin injected into the GA. These findings showed that combined gene therapy with both Ezrin targeting GA and L-periaxin targeting PN could be an effective treatment strategy for CMT4F.

### Ezrin was specifically expressed in myoblasts and their skeletal muscle fibers

**Fig. 2:**
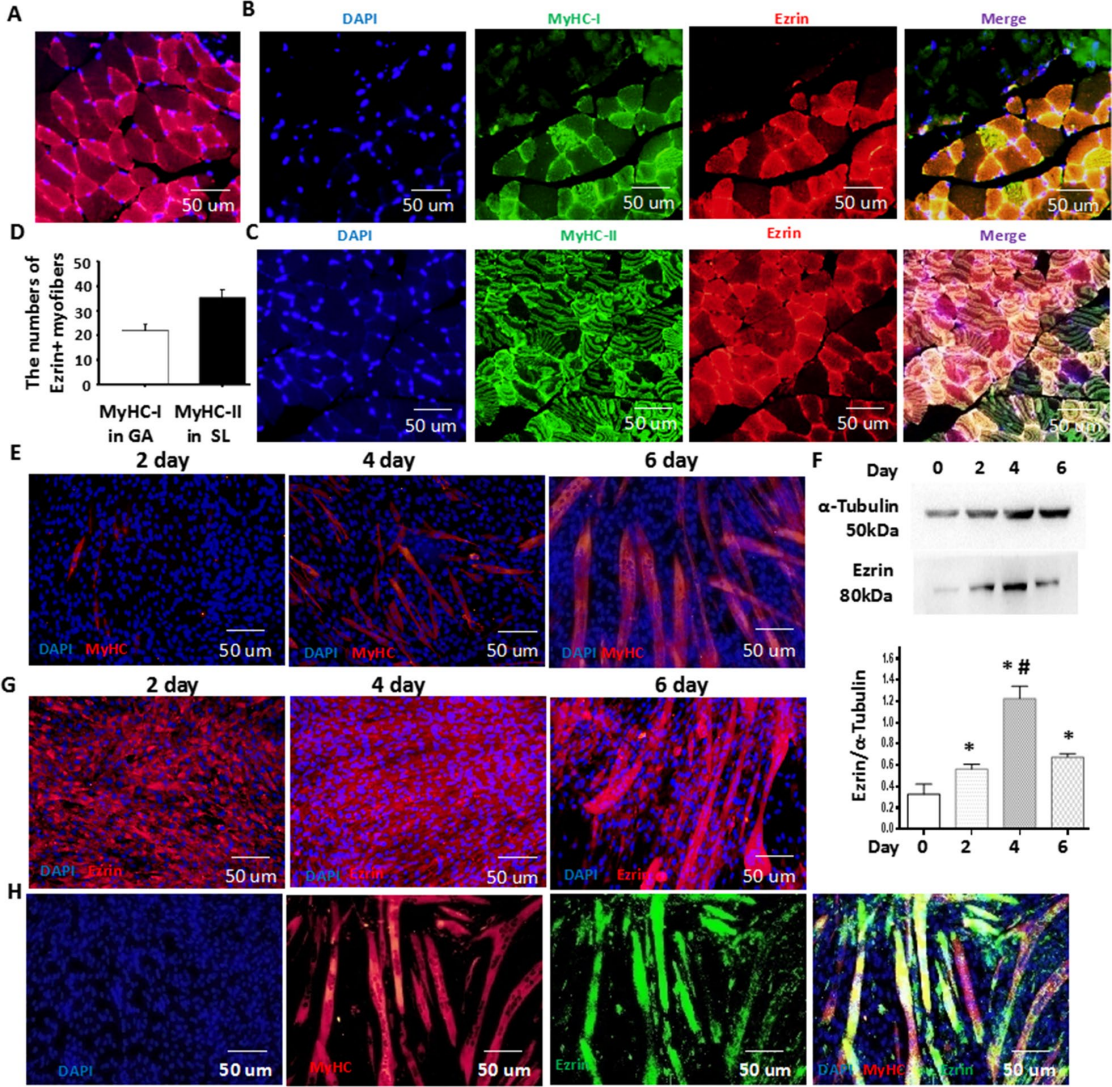
Ezrin was specifically expressed in myoblasts and their skeletal muscle fibers. **A** Typical image of Ezrin expression in the gastrocnemius muscle. Red fluorescence indicates Ezrin, and DAPI indicates the nucleus. **B** Typical image of Ezrin expression in soleus muscles. Red fluorescence indicates Ezrin; green fluorescence indicates MyHC-I; DAPI indicates the nucleus. **C** Typical image of Ezrin expression in the gastrocnemius muscle. Red fluorescence indicates Ezrin; green fluorescence indicates MyHC-I; DAPI indicates the nucleus. **D** Quantitative assay for Ezrin expression in muscle. **E** The C2C12 myoblast differentiation model was established. Red fluorescence indicates MyHC; DAPI indicates the nucleus. **F** Ezrin expression was detected by Western blot at 0, 2, 4 and 6 days after myoblast differentiation. A quantitative assay for Ezrin expression was performed 2, 4 and 6 days after myoblast differentiation. Three independent experiments were performed, n = 3, *P < *0.05* vs. 0 days; ^*#*^*P* < *0.05* vs. 2 days; ^*&*^*P* < *0.05* vs. 4 days. **G** Typical image of Ezrin expression in the differentiaited myoblasts at 2, 4 and 6 days. Red fluorescence indicates Ezrin; DAPI indicates the nucleus. **H** Typical image of the colocalization of MyHC and Ezrin at 6 days after myoblast differentiation. Red fluorescence indicates MyHC; green fluorescence indicates Ezrin; DAPI indicates the nucleus

To further confirm the role of Ezrin in skeletal muscle, we first detected whether Ezrin was expressed in gastrocnemius muscle, as shown in Fig. 2A, and myofibers partially expressed Ezrin. To distinguish the traits of Ezrin expression in different myofibers, including MyHC-I and MyHC-II, we showed that more MyHC-II myofibers were positive for Ezrin through double immunofluorescence staining, in addition to MyHC-I myofibers (Fig. 2B–D). Subsequently, to further confirm whether Ezrin was expressed in myoblast cells and differentiating myoblast cells, the expression of Ezrin during the process of C2C12 cell differentiation/fusion was analyzed by immunofluorescence staining and western blot. We found that Ezrin expression gradually increased during myoblast differentiation, reaching peak levels on day 4 of differentiation (Fig. 2E–G). In line with Fig. 1B, differentiated C2C12 myoblast cells and formed myotubes showed positive Ezrin expression (Fig. 2H). These results indicated that the specific expression of Ezrin in myoblasts and their skeletal muscle fibers could be involved in myoblast differentiation/fusion and muscle fiber specialization.

### Ezrin was involved in myoblast differentiation/fusion

To determine the effect of Ezrin on myoblast differentiation and fusion, we transfected C2C12 cells with adenovirus vectors carrying either Ezrin or shRNA-Ezrin for overexpression (Ad-Ezrin) or knockdown (Ad-shEzrin), respectively. Meanwhile, C2C12 cells transfected with adenovirus empty vector (Ad-Null) were used as a control. Assessment of the transfection efficiency revealed that following the application of 100 optimal multiplication of infection (MOI) with Ad-Ezrin or Ad-shEzrin, C2C12 myoblasts almost reached a confluence of 95% (Additional file 1: Figure. S2A-C). His-tag detection was further used to confirm the successful transfection of the adenovirus vector (Additional file 1: Figure. S2C-E), showing the high levels of His-tag in C2C12 myoblasts treated with either Ad-Ezrin or Ad-shEzrin. Furthermore, Ezrin protein levels were obviously increased in Ad-Ezrin-treated C2C12 myoblasts, while Ezrin levels were markedly decreased in Ad-shEzrin-treated C2C12 myoblasts (Additional file 1: Figure. S2C-E), indicating that the Ezrin gene was successfully overexpressed and knocked down.

**Fig. 3:**
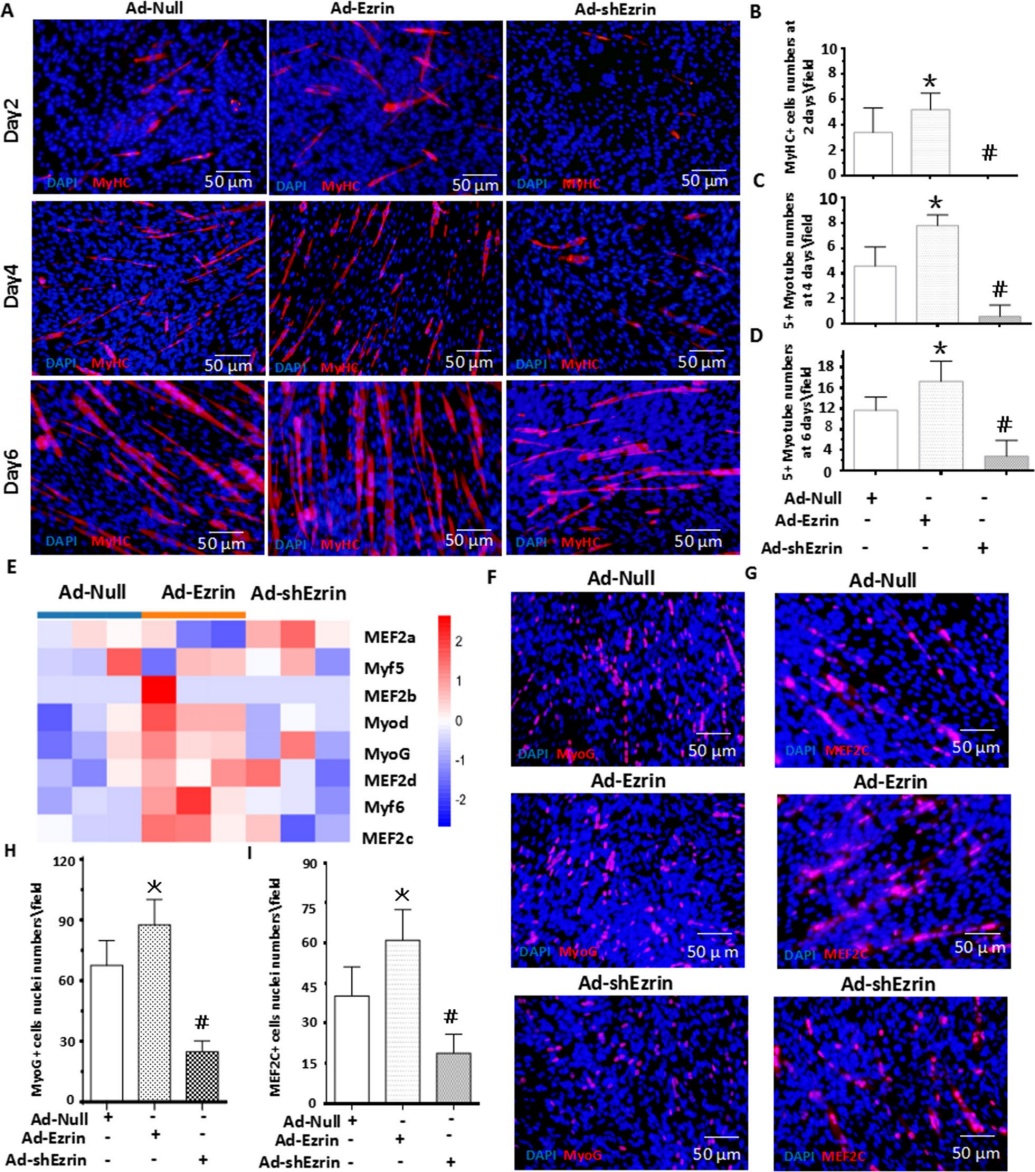
Ezrin was involved in myoblast differentiation/fusio. **A** Typical image of MyHC staining in differentiated C2C12 myoblasts treated with Ezrin overexpression or knockdown for 2, 4 and 6 days. **B**–**D** A quantitative assay for the number of MyHC + myotubes with more than 5 nuclei was performed 2, 4 and 6 days after myoblast differentiation after overexpression or knockdown of Ezrin. Three independent experiments were performed, n = 3, *P < *0.05* vs. Ad-Null; *#P* < *0.05* vs. Ad-Null. **E** RNA-seq analysis of myoblast differentiation in C2C12 myoblasts with overexpression or knockdown of Ezrin at 6 days. n = 3. **F**–**I** MyoG and MEF2c fluorescence staining and quantitative assay for myoblast differentiation. Red fluorescence indicates MyoG or MEF2c; DAPI indicates the nucleus. Three independent experiments were performed, n = 3, *P < *0.05* vs. Ad-Null; ^*#*^*P* < *0.05* vs. Ad-Null

The formation of myotubes is often judged by the fusion of differentiated myoblasts characterized by three or more nuclei in a myotube [15, 16]. Our results further showed that the number of MyHC + myotubes with either 3–5 or 5^+^ nuclei increased upon treatment with Ad-Ezrin in a time-dependent manner (Fig. 3A–D). Conversely, knockdown of Ezrin by shRNA not only obviously reduced the MyHC + cell number but also dramatically decreased the number of myotubes with either 3–5 or 5^+^ nuclei (Fig. 3A–D). These findings suggested that Ezrin could play a critical role in myoblast differentiation and fusion.

### Ezrin promoted myoblast differentiation/fusion in a manner dependent on low levels of L-periaxin

**Fig. 4:**
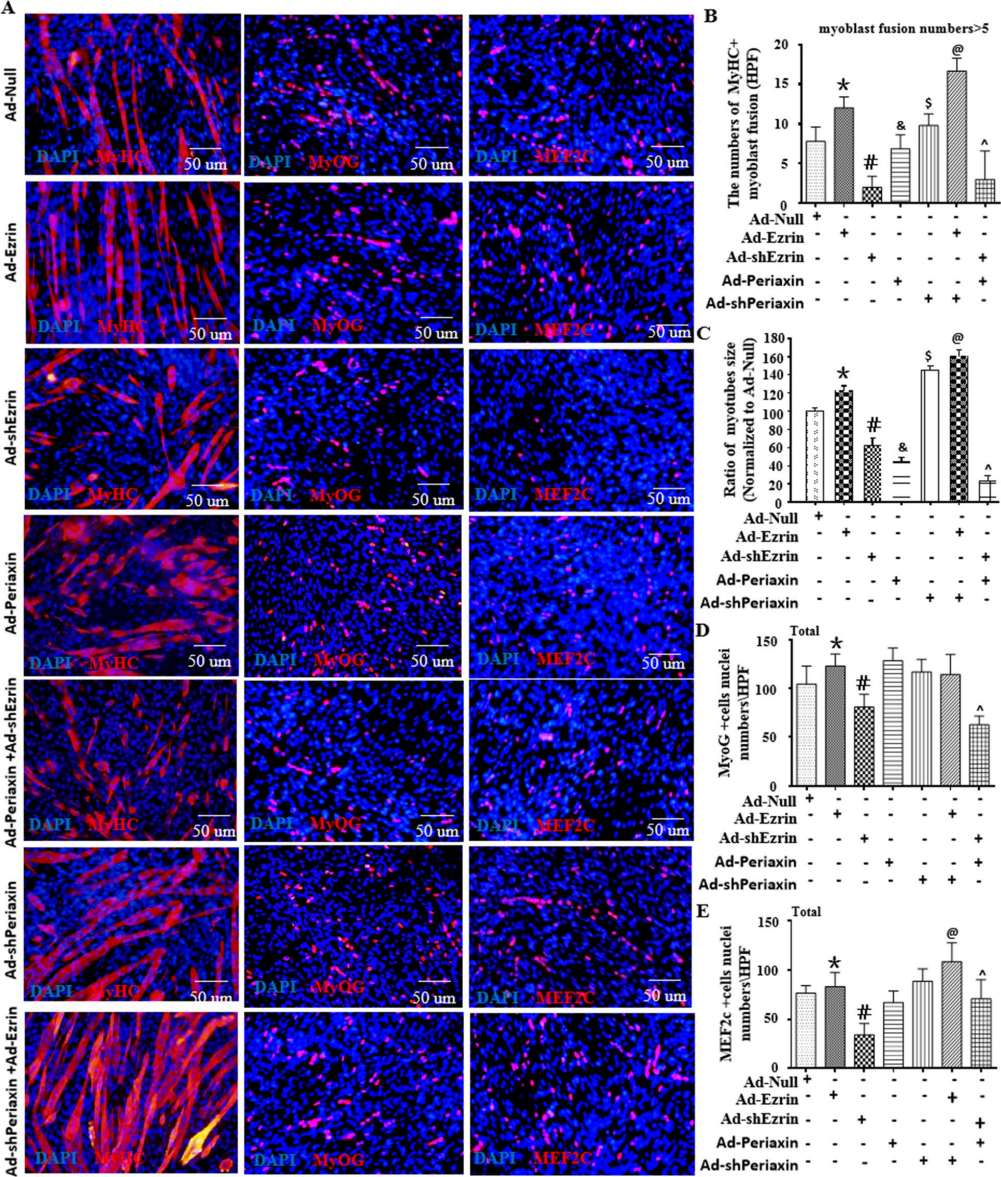
Low levels of L-periaxin were required for Ezrin to activate MyoG/MEF2C-mediated myoblast differentiation/fusion. **A** Typical image of MyHC, MyoG or MEF2c staining in differentiated C2C12 myoblasts with overexpression or knockdown of Ezrin with or without Ad-Periaxin or Ad-shPeriaxin at 6 days of differentiation. Red fluorescence indicates MyHC, MyoG or MEF2c; DAPI indicates the nucleus. **B** Ad-Ezrin, but not Ad-Periaxin, increased the numbers of MyHC + myotubes with 5^+^ myoblast fusion, as determined by a quantitative assay of myotubes, and the Ad-Ezrin effect was enhanced by knockdown of L-periaxin by shRNA in myoblasts. **C** Ad-Ezrin or Ad-shPeriaxin increased myotube size in MyHC-positive myotubes with 5^+^ myoblast fusion, while Ad-shEzrin or Ad-Periaxin decreased them as analyzed by quantitative assay of the ratio in myotube size normalized to the Ad-null group. Ad-shPeriaxin enhanced the effect of Ad-Ezrin on myotube size, while Ad-Periaxin further deteriorated the inhibitory effect of Ad-shEzrin on myotube size. **D**, **E** Quantitative assay for MEF2c + or MyoG + nuclei numbers in the above images. Three independent experiments were performed, n = 3, *P < *0.05* vs. Ad-Null; ^*#*^*P* < *0.05* vs. Ad-Null; ^*&*^*P* < *0.05* vs. Ad-Null; ^*$*^*P* < *0.05* vs. Ad-Null; ^*@*^*P* < *0.05* vs. Ad-Ezrin*; ^P* < *0.05* vs. Ad-shEzrin

To confirm whether L-periaxin/Ezrin’s independence and interaction are involved in myoblast differentiation and fusion in vitro, simultaneous transfection experiments for overexpression or knockdown of L-periaxin and Ezrin were performed. As shown in Fig. 4, we found that Ad-Periaxin did not reverse the inhibitory effects of Ad-shEzrin on myoblast differentiation and fusion in vitro but further reduced the length of myotubes (Fig. 4A–C and Additional file 1: Figure S4A-B). Of interest, Ad-Periaxin alone shortened the length of myotubes during myoblast differentiation and fusion, while Ad-shPeriaxin alone increased myotube area. More importantly, Ad-shPeriaxin enhanced the beneficial effects of Ad-Ezrin on myoblast differentiation and fusion in vitro*,* especially in increasing the length of myotubes (Fig. 4A–C and Additional file 1: S4A-B). Indeed, in the process of myoblast differentiation and fusion, the sixth day was the most typical. After that, the length and area of myotubes did not increase significantly but decreased. Similarly, the expression of Ezrin obviously decreased on the sixth day, with the highest expression levels of Ezrin on the 4th day of early differentiation (Fig. 2F), and L-periaxin expression was transiently and substantially increased on the 6th day of late differentiation (Additional file 1: Figure S5A–C), indicating that a sudden increase in endogenous expression of L-periaxin could limit the length and area of myotubes accompanied by a decrease in Ezrin during myoblast differentiation/fusion.

### Low levels of L-periaxin were required for Ezrin to activate MyoG/MEF2C-mediated myoblast differentiation

MyoG and MEF2C play an important role in the early and late differentiation of myoblasts, respectively [20]. Using RNA-seq to analyze the changes in genes in myoblasts treated with Ad-Ezrin or Ad-shEzrin, we found that the expression levels of MyoD, MyoG and MEF2c, as classical regulators of myogenic differentiation, were obviously increased in myoblasts treated with Ad-Ezrin; conversely, their expression levels were decreased in the Ad-shEzrin group compared with the Ad-null group (Fig. 3E and Additional file 1: Figure S1B). Further immunofluorescent staining showed that the positive numbers of MyoG and MEF2C following treatment with Ad-Ezrin were markedly increased (Fig. 3F, I), which was consistent with the highest expression levels of Ezrin on the 4th day of early differentiation (Fig. 2F). Of interest, instead of negating the role of Ad-Ezrin, Ad-shPeriaxin enhanced the role of Ad-Ezrin in increasing the number of MEF2C + nuclei (Fig. 4A–E). Conversely, Ezrin knockdown by shRNA dramatically decreased the positive numbers of MyoG and MEF2C in myoblasts (Fig. 3F–I). Meanwhile, Ad-Periaxin reversed the inhibitory effects of Ad-shEzrin on MEF2C-positive numbers but not on MyoG (Fig. 4A, D and E). Therefore, a proper match of Ezrin and periaxin at the protein level is involved in myogenic differentiation.

### Ezrin was involved in myofiber specialization

**Fig. 5:**
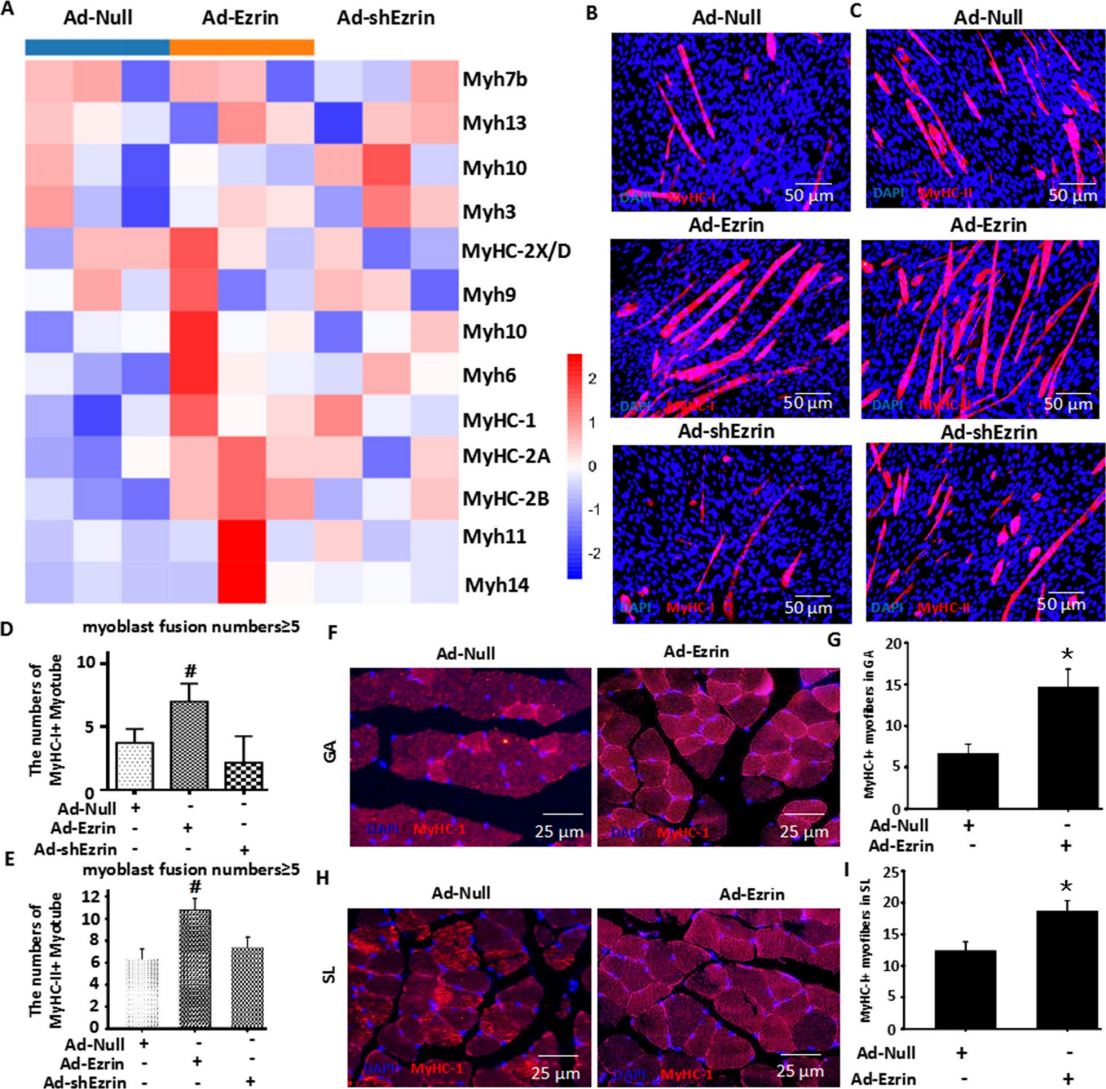
Ezrin is involved in myofiber specialization. **A** RNA-seq analysis of myofiber types in C2C12 myoblasts with overexpression or knockdown of Ezrin at 6 days. n = 3. **B**–**E** MyHC-I and MyHC-II fluorescence staining **B**–**C** and quantitative assay **D**, **E** for myoblast differentiation. Red fluorescence indicates MyHC-I (B: NOQ) or MyHC-II (C: MY32); DAPI indicates the nucleus. Three independent experiments were performed, n = 3, *P < *0.05* vs. Ad-Null; ^*#*^*P* < *0.05* vs. Ad-Null. (**F–I**) In vivo assay for MyHC-1 expression in gastrocnemius (GA) and soleus (SL) muscles following the local injection of Ad-Ezrin. Red fluorescence indicates MyHC-I (**F, G**: NOQ); DAPI indicates the nucleus. n = 6, *P < *0.05* vs. Ad-Null

Because the types of muscle fibers are closely related to the contraction and metabolic function of skeletal muscle [14], using RNA-seq to analyze the traits of myofibers formed by myoblast differentiation, we found that the expression levels of MyHC-1, MyHC-2a, MyHC-2b and MyHC-2X were substantially increased in Ad-Ezrin-treated myoblasts (Fig. 5A). Further immunofluorescent staining showed that the numbers of MyHC-1- or MyHC-2-positive myotubes were obviously increased in Ad-Ezrin-treated myoblasts (Fig. 5B–D). However, knockdown of Ezrin by shRNA slightly reduced the number of MyHC-1-positive myotubes while slightly increasing the number of MyHC-2-positive myotubes, but the difference was not significant compared with that in the Ad-Null group (Figs. 2B, 5B–E), similar to the RNA-seq results shown in Additional file 1: Figure S5A. To further confirm the effects of Ezrin on muscle fiber type composition, local injection of Ad-Ezrin into the GA and soleus muscles (SL) significantly increased MyHC-1-positive myofiber numbers in the GA and SL (Fig. 5D–I). Therefore, Ezrin could be involved in the regulation of myofibers in vivo and in vitro.

### Ezrin regulated myoblast differentiation and fusion through the PKA signaling pathway

**Fig. 6:**
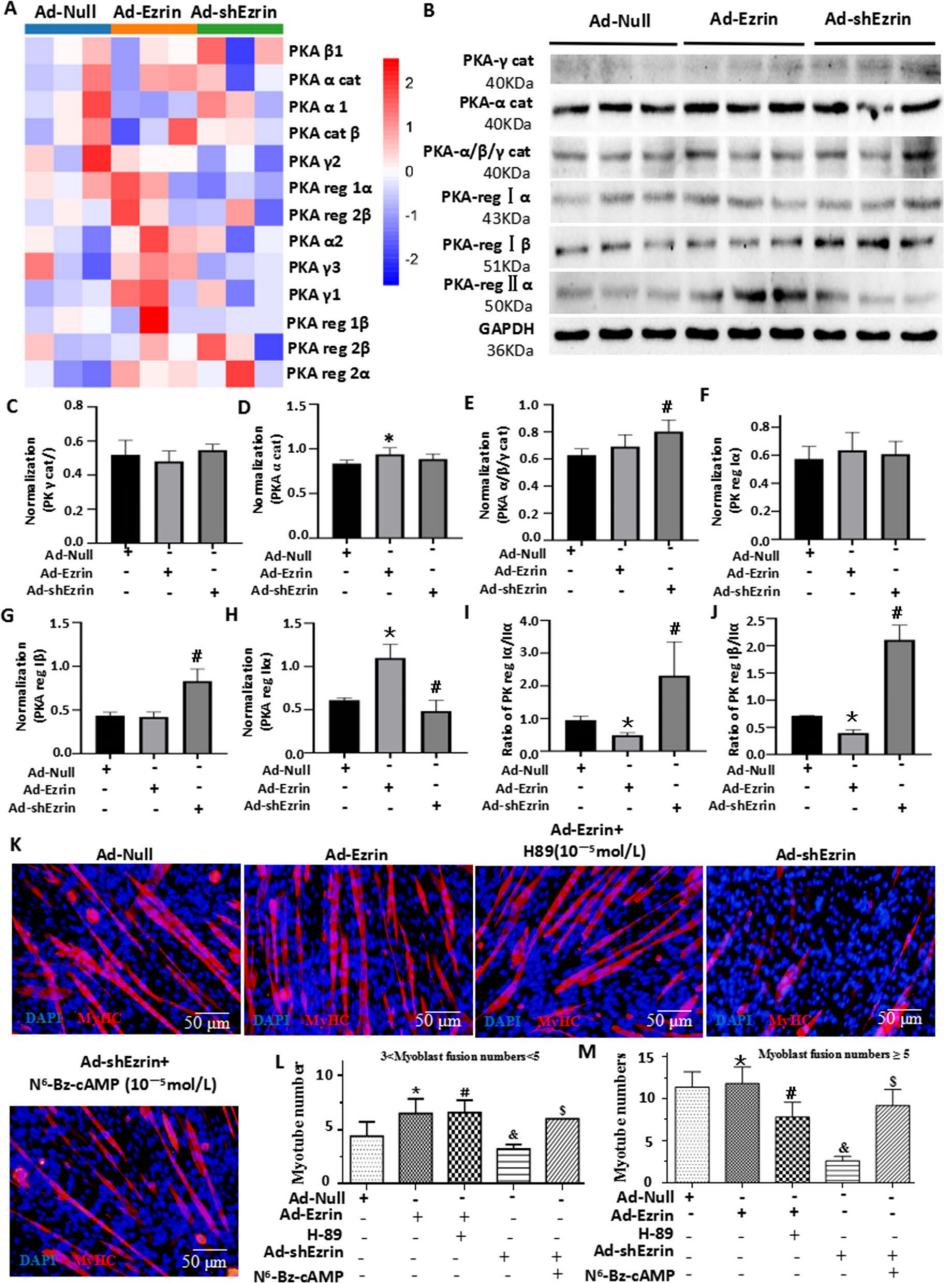
Ezrin promoted myoblast differentiation and fusion through the PKA-MyoG/MEF2C signaling pathway. **A** RNA-seq analysis of the PKA signaling pathway in C2C12 myoblasts with overexpression or knockdown of Ezrin at 6 days. n = 3. **B** Western blot for the indicated proteins in myoblasts treated with Ezrin overexpression or knockdown for 6 days. **C**–**J** Quantitative assays for the indicated proteins were performed 6 days after myoblast differentiation following Ezrin overexpression or knockdown. Three independent experiments were performed, n = 3, *P < *0.05* vs. Ad-Null; ^*#*^*P* < *0.05* vs. Ad-Null. **K** Typical image of MyHC staining in differentiated C2C12 myoblasts treated with Ad-Ezrin with or without H-89 (10^–5^ mol/L) or Ad-shEzrin with or without N^6^-Bz-cAMP (10^–5^ mol/L). Red fluorescence indicates MyHC; DAPI indicates the nucleus. **L**, **M** Quantitative assays for the number of MyHC + myotubes with 3–5 or more than 5 nuclei were performed six days after myoblast differentiation. Three independent experiments were performed, n = 3, *P < *0.05* vs. Ad-Null; ^*#*^*P* < *0.05* vs. Ad-Ezrin*; *^*&*^*P* < *0.05* vs. Ad-Null; ^*$*^*P* < *0.05* vs. Ad-shEzrin

Myoblast differentiation and fusion are involved in alterations in both PKA activity and the PKAreg I/II ratio [8, 9]. Using RNA-seq and western blotting to analyze the changes in Ad-Ezrin- or Ad-shEzrin-treated myoblasts, we found that Ad-Ezrin did not alter the levels of PKA-γ cat, PKA reg Iα and Iβ but significantly increased the levels of PKA-α cat and PKA reg II α, PKA cat α, PKA reg Iα and PKA reg IIα, causing a decrease in the PKA reg I/II ratio. In contrast, knockdown of Ezrin by shRNA did not change the levels of PKA-α cat, PKA-γ cat and PKA reg Iα but obviously caused increased levels of PKA reg Iβ and PKA cat α/β/γ and decreased levels of PKA reg IIα, leading to an increased PKA reg I/II ratio (Fig. 6A–J). Meanwhile, a PKA inhibitor, H-89, abolished the role of Ad-Ezrin in promoting myoblast differentiation and fusion. In contrast, the PKA activator N^6^-Bz-cAMP reversed the inhibitory effects of Ad-shEzrin on myoblast differentiation and fusion (Fig. 6K–M). Therefore, the role of Ezrin in myoblast differentiation and fusion could be involved in the PKA signaling pathway.

### Ezrin promoted myoblast differentiation and fusion through the PKA-MyoG/MEF2C signaling pathway

Using immunofluorescent staining, we further found that the numbers of MyoG- or MEF2C-positive nuclei were substantially increased in Ad-Ezrin-treated myoblasts, which was obviously abrogated by the PKA inhibitor H-89 (Additional file 1: Figure S7A–F). In contrast, these nuclei numbers were markedly decreased in Ad-shEzrin-treated myoblasts, and the inhibitory effects could be reversed by a PKA activator, N^6^-Bz-cAMP (Additional file 1: Figure. S7A–F and Figure. S8A–F). Meanwhile, western blotting showed that Ad-Ezrin increased the nuclear levels of MyoG and MEF2C, while Ad-shEzrin decreased their levels. Furthermore, the increase in the nuclear levels of MyoG and MEF2C induced by Ad-Ezrin could be significantly abolished by H-89. Similarly, the effect of Ad-shEzrin on MyoG and MEF2C was partially reversed by N^6^-Bz-cAMP (Additional file 1: Figure S7G–I). These results indicated that Ezrin participated in C2C12 myoblast differentiation and fusion through the PKA-MyoG/MEF2C signaling pathway.

### NFAT signaling is involved in the regulation of myoblast differentiation/fusion mediated by Ezrin

**Fig. 7:**
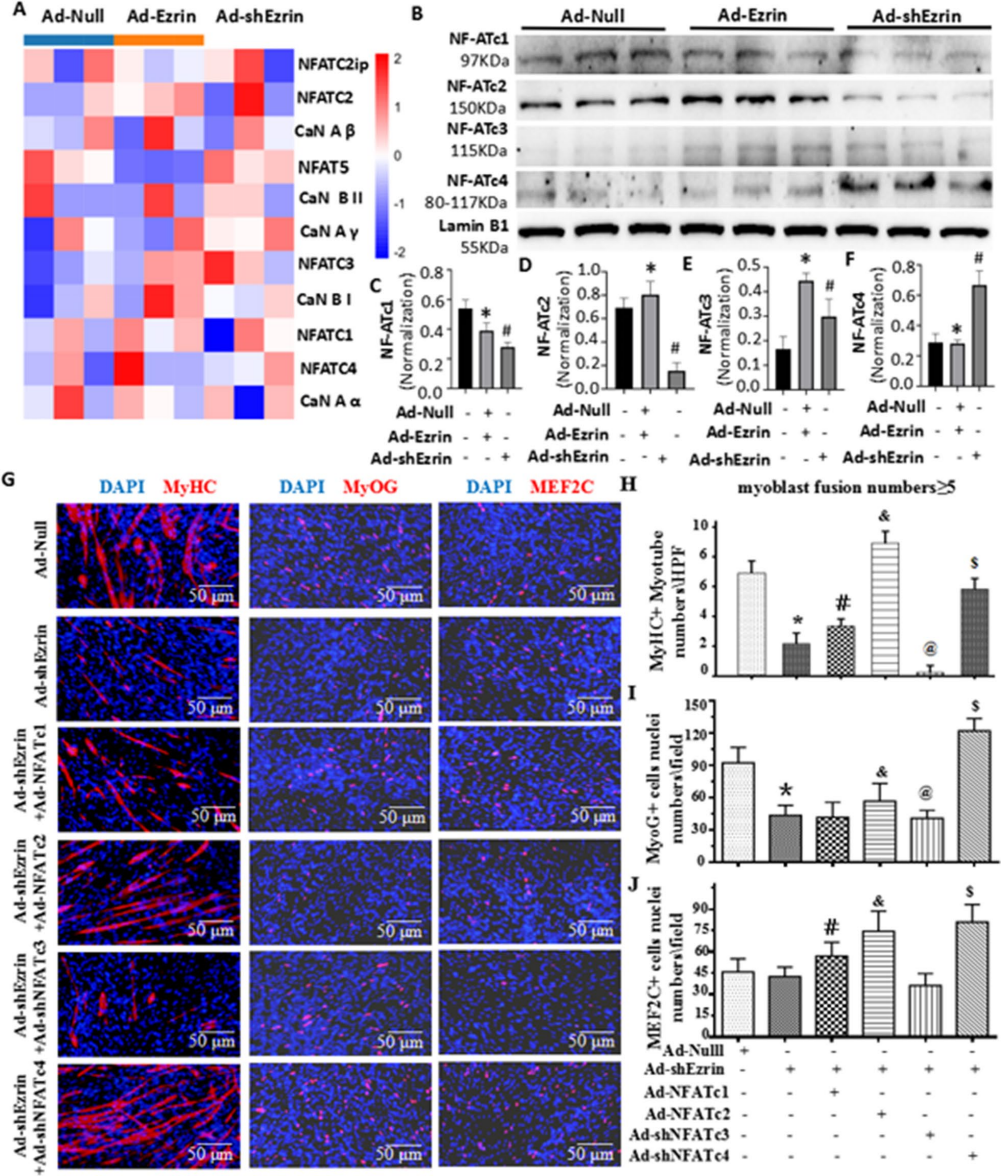
NFAT signaling is involved in the regulation of myoblast differentiation/fusion mediated by Ezrin. **A** RNA-seq analysis of the CaN-NFAT signaling pathway in C2C12 myoblasts with overexpression or knockdown of Ezrin at 6 days. n = 3. **B** Western blot of NFATc1-c4 proteins in myoblasts treated with Ezrin overexpression or knockdown for 6 days. **C**–**F** Quantitative analysis of NFATc1-c4 protein expression was performed 6 days after myoblast differentiation following Ezrin overexpression or knockdown. Three independent experiments were performed, n = 3, *P < *0.05* vs. Ad-Null; ^*#*^*P* < *0.05* vs. Ad-Null. **G** Typical image of MyHC, MyoG or MEF2c staining in differentiated C2C12 myoblasts treated with Ad-shEzrin with or without Ad-NFATc1, Ad-NFATc2, Ad-shNFATc3 and Ad-shNFATc4. Red fluorescence indicates MyHC, MyoG or MEF2c; DAPI indicates the nucleus. **H**–**J** Quantitative assay for MyHC + myotubes and MEF2c + or MyoG + nuclei numbers in the above images. Three independent experiments were performed, n = 3, *P < *0.05* vs. Ad-Null; ^*#*^*P* < *0.05* vs. Ad-shEzrin; ^*&*^*P* < *0.05* vs. Ad-shEzrin; ^*$*^*P* < *0.05* vs. Ad-shEzrin

NF-κB plays a crucial role during myoblast differentiation/fusion, especially in myofiber specification [21–27]. RNA-seq and western blotting were used to analyze changes in Ad-Ezrin- or Ad-shEzrin-treated myoblasts. We found that Ad-Ezrin increased the nuclear levels of NFATc1/c2 while decreasing NFATc3/c4 nuclear levels in myoblasts. Conversely, Ad-shEzrin reduced NFATc1/c2 nuclear levels while increasing NFATc3/c4 nuclear levels (Fig. 7A–F, Additional file 1: Figure S9A-B). We further found that the overexpression of NFATc2 almost completely reversed the role of Ad-shEzrin in reducing the numbers of MyHC-positive myotubes with more than nuclei (Fig. 7G, H). Knockdown of NFATc3 by shRNA markedly deteriorated the inhibitory effects of Ad-shEzrin on myoblast differentiation and fusion. However, knockdown of NFATc4 by shRNA significantly abolished the inhibitory effects of Ad-shEzrin on myoblast differentiation/fusion (Fig. 7G, H).

The roles of MyoG and MEF2C in the initiation and later stages of myoblast differentiation, respectively, have been considered [28]. We found that either Ad-NFATc2 or Ad-shNFATc4 obviously abolished the inhibitory role of Ad-shEzrin in decreasing the number and percentage of MyoG + and MEF2C + nuclei in fewer than 3-nucleus cells and more than 3-nucleus myotubes (Figure 7G, I–J, Additional file 1: Figure S10A-D). In addition, Ad-NFATc1 partially reversed the number of MEF2C-positive nuclei in 3-nuclei^+^ myotubes. However, Ad-shNFATc3 did not change the number of MEF2C- or MyoG-positive nuclei in 3-nuclei^+^ myotubes. Therefore, Ezrin is involved in myoblast differentiation and fusion through NFATc2/c4-MyoG/MEF2C, at least in part.

### NFAT signaling is involved in the regulation of myofiber specialization mediated by Ezrin

**Fig. 8:**
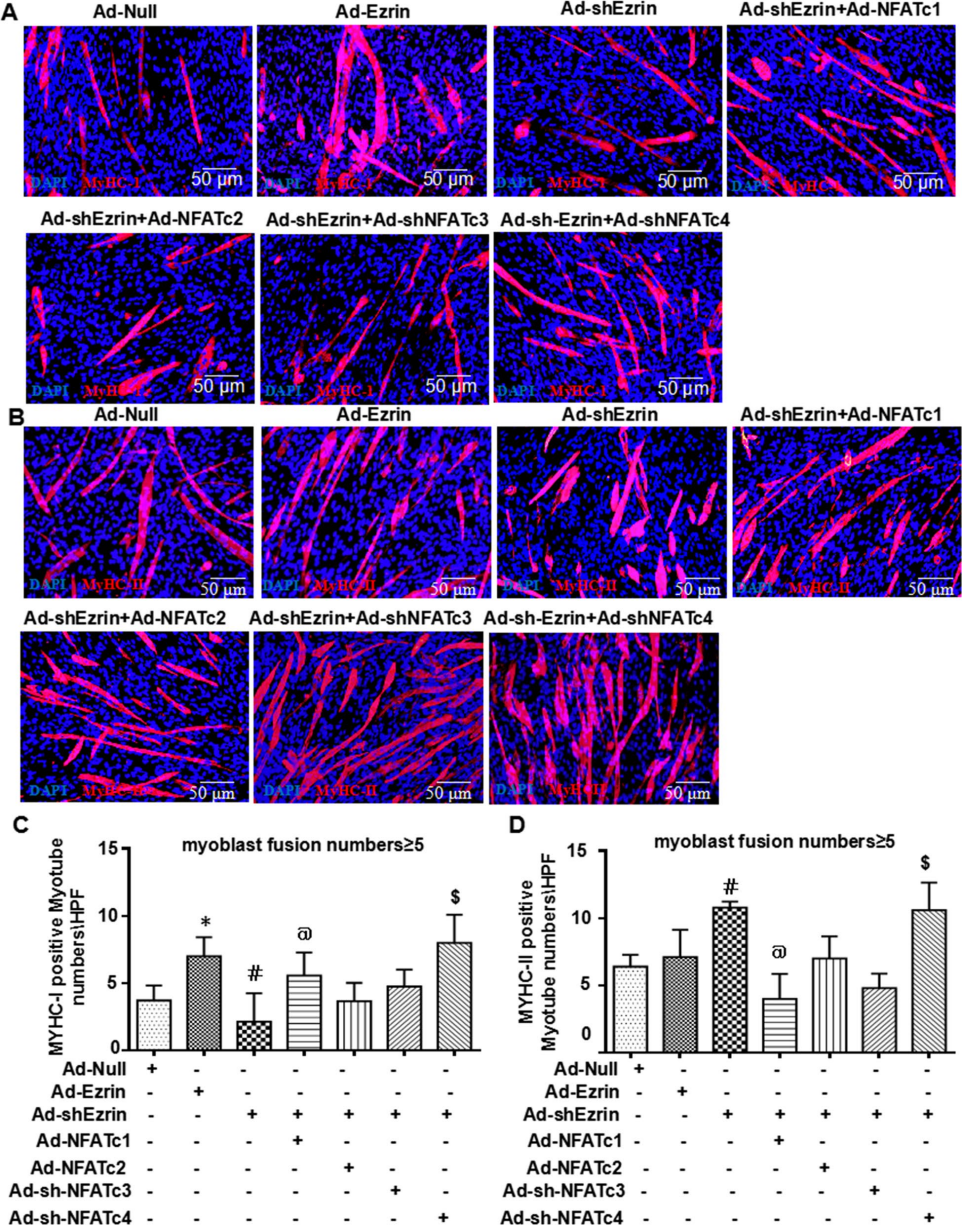
NFAT signaling is involved in the regulation of myofiber specialization mediated by Ezrin. **A**, **B** Typical image of MyHC-I (NOQ) or MyHC-II (MY32) staining in differentiated C2C12 myoblasts treated with Ad-shEzrin with or without Ad-NFATc1, Ad-NFATc2, Ad-shNFATc3 and Ad-shNFATc4. Red fluorescence indicates MyHC-I **A** or MyHC-II **B**; DAPI indicates the nucleus. **C**, **D** Quantitative assay for MyHC-I-positive and MyHC-II-positive myotubes in the above images. Three independent experiments were performed, n = 3, *P < *0.05* vs. Ad-Null; ^*#*^*P* < *0.05* vs. Ad-Null; ^*$*^*P* < *0.05* vs. Ad-shEzrin; ^*@*^*P* < *0.05* vs. Ad-shEzrin

Often, muscle fibers are divided into slow and fast muscles; MyHC-1 is the main component of the former, and MyHC-2a, MyHC-2b and MyHC-2X are the main components of the latter [29, 30]. Similar to the RNA-seq results (Fig. 5A), real-time PCR showed that Ad-Ezrin typically increased the expression of MyHC-2a and MyHC-2b. More importantly, the increase in MyHC-2a- and MyHC-2b-mediated Ad-shEzrin expression was obviously abolished by Ad-NFATc1 or Ad-NFATc2 (Additional file 1: Figure S11A–C), respectively. Meanwhile, Ad-shEzrin-induced MyHC-2a expression could be enhanced by Ad-shNFATc3 or Ad-shNFATc4. However, Ad-shNFATc3 enhanced the effect of Ad-shEzrin on MyHC-2b expression, while Ad-shNFATc4 reversed this effect (Additional file 1: Figure S11A–C). Further immunofluorescent staining showed that Ad-Ezrin increased the numbers of MyHC-1- and MyHC-2-positive myotubes, while Ad-shEzrin increased the numbers of MyHC-2-positive myotubes and decreased the numbers of MyHC-1-positive myotubes (Fig. 8A–D). The Ad-shEzrin effects could be canceled by Ad-NFATc1, Ad-NFATc2 or Ad-shNFATC3. Therefore, Ezrin-mediated MyHC-1/2-positive myofiber formation is involved in NFAT signaling.

## Discussion

In this study, we made three novel observations. First, we found that Ezrin was a candidate target for the treatment of muscle atrophy by promoting the regeneration and repair of the damaged gastrocnemius muscle induced by peroneal nerve injury, especially in CMT4F-associated muscle atrophy. Second, high levels of Ezrin and low levels of L-periaxin cooperatively controlled the length and size of myotubes. Finally, Ezrin significantly controlled myoblast differentiation and fusion through the PKA-NFAT-MyoD/MEF2C signaling pathway.

**Fig. 9:**
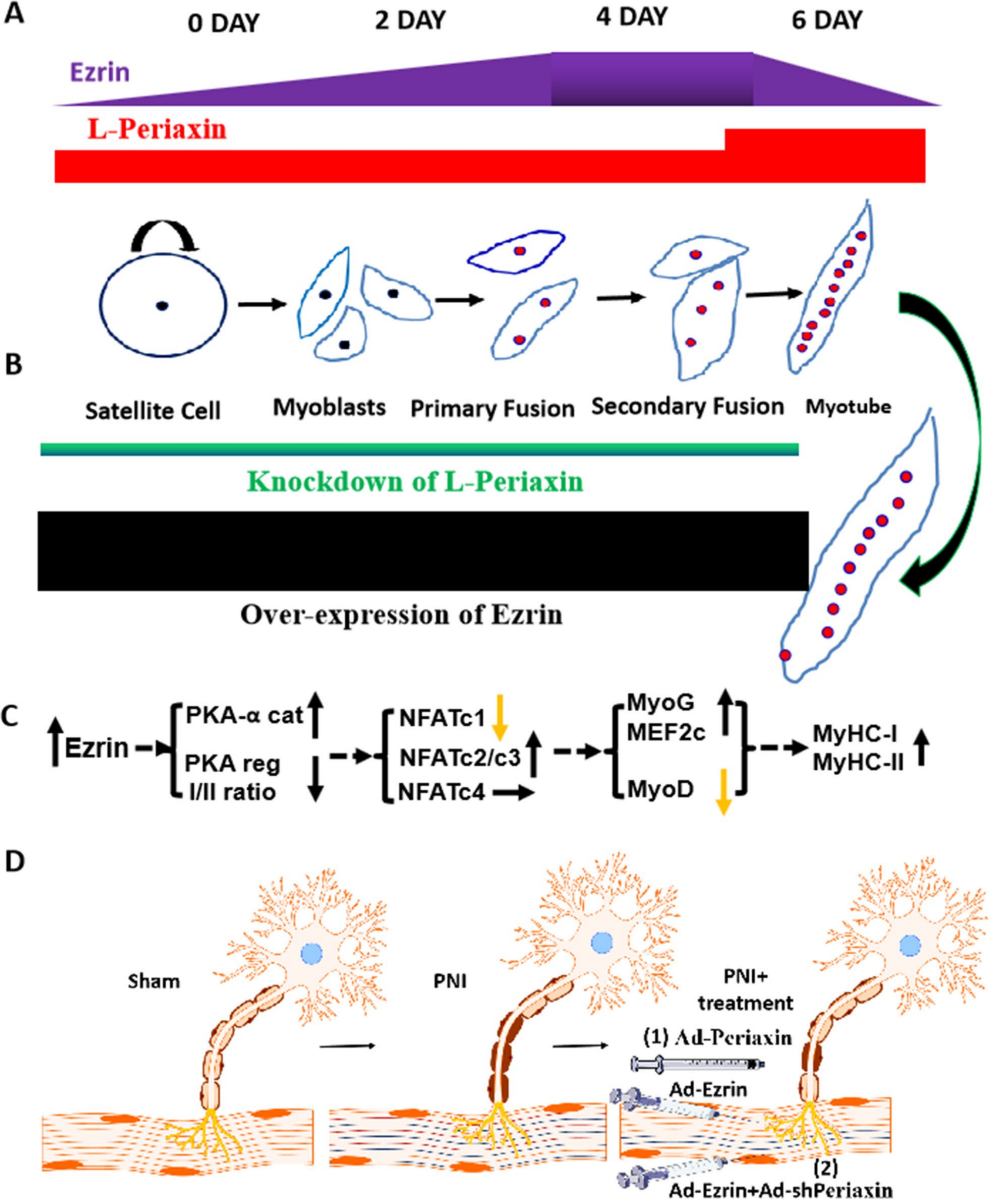
Working Model: The spatiotemporal matching pattern of Ezrin/Periaxin was involved in muscle repair in a peroneal nerve injury model. Instantaneous L-periaxin expression was highest on the 6th day, while Ezrin expression peaked on the 4th day during myoblast differentiation/fusion (**A**). Ezrin promoted myoblast differentiation/fusion, myotube length and size, and myofiber specialization. L-periaxin acted as a brake for myotube length and size, negatively regulating the effect of Ezrin on myotube formation (**A, B**). The effects of Ezrin were related to the activated PKA-NFAT-MyoD/MEF2C signaling pathway (**C**). Combined gene therapy with Ezrin and L-periaxin contributed to muscle repair in a peroneal nerve injury model (**D**), providing a novel therapeutic strategy for the treatment of nerve injury, especially CMT4F-associated muscle atrophy

Published data have shown that periaxin is one of the scaffold proteins specifically expressed in Schwann cells, playing an important role in the formation and stability of the myelin sheath and the control of axon size [4–6]. Clinically, deletion or mutation of the periaxin gene can cause the demyelinating CMT4F-associated muscle atrophy [4, 5]. In the present study, a peroneal nerve injury (PNI) model was successfully established to partially mimic CMT4F-associated muscle atrophy. One local muscle injection of Ad-Ezrin, not Ad-Periaxin, dramatically increased the numbers of MyHC-I- and MyHC-II-positive fibers characterized by finer fibers, leading to the partial improvement in gastrocnemius muscle atrophy induced by PNI, indicating that gene therapy targeting Ezrin alone could be insufficient for imhereditary muscle atrophy. PNI-induced muscular atrophy showed the traits of demyelination and associated muscular atrophy [31], similar to the changes caused by L-periaxin deletion or mutation [4, 5] and Ezrin-mediated L-periaxin self-association inhibition [6]. Actually, it is different because further evidence that local muscle injection of Ad-Ezrin combined with local treatment of Ad-shPeriaxin injection into PNI-injured gastrocnemius muscle could not only increase the number of muscle fibers but also recover its size to a relatively normal level in vivo. The in vitro results also showed that the combination of Ad-Ezrin and Ad-shPeriaxin better promoted myoblast differentiation/fusion and myotube length and size (Fig. 1). Thus far, muscular atrophy of CMT4F has not been solved, but problems such as nerve demyelination have been solved. The novel strategy of simultaneous nerve and muscle repair was performed, showing that local muscle injection of Ad-Ezrin combined with local treatment of Ad-Periaxin into the peroneal nerve better restored muscle fiber number and size to a near normal level compared with local treatment of combined injection of Ad-Ezrin and Ad-shPeriaxin into the gastrocnemius muscle (Fig. 1). In summary, under the premise of considering hereditary muscular atrophy disease, especially L-periaxin deletion or mutation, combined gene therapy targeting Ezrin with L-periaxin could be a novel and potential clinical strategy for the treatment of muscular atrophy caused by a variety of pathological factors, especially nerve injury (Fig. 9).

Of interest, L-periaxin plays an important role in the formation and stability of the myelin sheath and the control of axon size [4, 5]. Overexpression of Ezrin in sciatic nerve injury could repair the injured myelin sheath. Furthermore, the interaction between L-periaxin and Ezrin adopted a close form to achieve protein accumulation and to help the maintenance of the myelin sheath [6]. Herein, for the first time, L-periaxin expression showed transient and substantial increases on the 6th day of late differentiation with unchanged expression in the early stage of differentiation, showing a specific role in limiting myotube length and size (Fig. 4). The evidence of these findings has been supported by the present results that L-periaxin overexpression did not affect myoblast differentiation/fusion but reduced the length and size of myotubes, while knockdown of L-periaxin by shRNA enhanced their changes (Fig. 4). Of interest, the combination of local muscle injection of Ad-Ezrin with local incubation of Ad-Periaxin within the peroneal nerve effectively recovered muscle fiber number and size to near normal levels. These results indicated that the L-periaxin could act as a different player in nerves and muscles, contributing to controlling myotube length and size to match the diameter size of the axon mediated by the L-periaxin (Fig. 9).

In fact, Ezrin expression showed the highest levels on the 4th day of early differentiation accompanied by a transient and substantial increase in L-periaxin on the 6th day of late differentiation (Fig. 2 and Additional file 1: Figure S5). The function of Ezrin in myoblast differentiation/fusion was further confirmed by the findings that Ad-Ezrin increased the length and size of myotubes, while Ad-shEzrin reduced these changes. Notably, Ad-Ezrin-mediated effects on promoting myoblast differentiation/fusion were obviously abolished by Ad-Periaxin. Conversely, knockdown of L-periaxin enhanced Ad-Ezrin-induced myotubes or myofibers to become longer and larger in vitro and in vivo*.* More importantly, combined injection of Ad-Ezrin with Ad-shPeriaxin into GA showed effective recovery of muscle fiber number and size, close to a relatively normal level (Figs. 1, 4). Therefore, the matching pattern of Ezrin and L-Periaxin expression could control not only the differentiation/fusion of myoblasts but also the length and size of myotubes (Fig. 9).

There have been many studies on the role and mechanism of Ezrin and L-periaxin in the formation and maintenance of the neural myelin sheath [4–6]. The present study showed that Ezrin plays an important role in myoblast differentiation and fusion compared with L-periaxin. To this end, little attention has been given to the mechanism of Ezrin in myoblast differentiation and fusion. In contrast to the role of PKA and associated Myf-5 and MyoD expression inhibition [21, 29, 32, 33], overexpression of Ezrin in myoblasts did not show any apparent changes in the expression of Myf-5 and MyoD but increased MyoG- and MEF2C-positive nuclei numbers(Fig. 3). Overexpression of Ezrin markedly increased PKA reg IIα levels, leading to a decreased PKA reg I/II ratio, accompanied by an acceleration of myoblast differentiation and fusion. Conversely, Ad-shEzrin resulted in a higher PKA reg I/II ratio, and the inhibitory effects induced by Ad-shEzrin on myoblast differentiation/fusion could be reversed by the PKA activator. Furthermore, the PKA activator almost completely recovered the number of MyoG- or MEF2C-positive myotubes mediated by Ad-shEzrin (Fig. 6). Therefore, Ezrin could promote myoblast differentiation/fusion via the PKA-MyoG/MEF2C signaling pathway (Fig. 9).

Existing data have shown that NFATs are crucial players in myoblast differentiation and fusion, especially myotube specification [21–27]. Moreover, NFAT activities are frequently regulated by the PKA-calcineurin signaling pathway during cell differentiation [34–37]. Indeed, NFATc2 primarily controls myoblast recruitment and myoblast fusion [16, 38–40]. NFATc3 promoted myoblast differentiation and fusion, while NFATc4 inhibited it [33, 41–45]. In addition to regulating the specialization of muscle fibers, NFATc1 can regulate myoblast fusion by promoting NFATc2 expression [46]. Herein, Ezrin overexpression reduced the levels of nuclear NFATc1 in myoblasts, but as expected, it reduced the levels of nuclear NFATc2. Meanwhile, Ad-Ezrin treatment obviously increased the levels of nuclear NFATc3 in myoblasts but did not change the nuclear NFATc4 levels. Of interest, knockdown of Ezrin by shRNA markedly decreased the nuclear levels of both NFATc1 and NFATc2 while increasing the nuclear levels of NFATc3/c4, leading to partial, but not complete, inhibition of myoblast differentiation/fusion and MyHC-I-positive myotube formation, which was attributed to the compensatory changes in NFATc3 caused by Ezrin knockdown. We further found that the application of Ad-NFATc2 or Ad-shNFATc4 could restore the above effect mediated by Ad-shEzrin (Fig. 7). However, Ad-NFATc1 partially reversed the Ad-shEzrin-mediated inhibitory effect. In contrast, knockdown of NFATc3 by shRNA deteriorated the inhibitory role of Ad-shEzrin. Therefore, altered NFATs signaling by Ezrin affected myoblast differentiation/fusion and MyHC-I myotube formation, especially NFATc2/c3 (Fig. 9).

Published data have shown that MEF2C and MyoD are involved in the regulation of MyHC-1- or MyHC-2a-positive myofiber specialization mediated by NFATc1 and NFATc3 [27, 36, 37, 40]. Herein, accompanied by an increase in nuclear NFATc3, Ad-Ezrin treatment substantially increased MEF2C levels within the nucleus, resulting in an obvious increase in MyHC-1 mRNA expression and positive myofibers. In contrast to the role of NFATc2 alone in regulating MyHC-2a expression [25], myoblasts treated with Ad-Ezrin showed the integrative traits of both an increase in NFATc2 and a decrease in NFATc1, causing unchanged MyHC-2a expression. In line with the evidence that NFATc4 mainly contributes to fast muscle fiber formation characterized by MyHC-2a, MyHC-2b and MyHC-2X [25, 39], Ad-Ezrin did not alter the number of MyHC-2-positive myofibers (Fig. 8), most likely due to the unchanged nuclear levels of NFATc4. Conversely, Ad-shEzrin treatment markedly increased MyoD expression while reducing nuclear MEF2C levels within myoblasts, leading to a decrease in MyHC-1-positive myofibers and an increase in MyHC-2-positive myofibers, which could be abolished by Ad-shNFATc3/c4 or Ad-NFATc1/c2. These results demonstrated that Ezrin could participate in myofiber specialization through the NFAT-MEF2C signaling pathway (Fig. 9).

In particular, although this study provides a new possible treatment strategy for muscular atrophy, especially CNT4F-related muscular atrophy, this study uses mice and C2C12 myoblast cfell lines from mice as experimental materials. Considering that there are differences in skeletal muscle fiber types and metabolism between humans and mice [7], and whether Ezrin/Periaxin have similar therapeutic value in humans, more detailed and in-depth studies should be carried out in large animals and human-derived myoblast cell lines in the future to confirm their therapeutic value and convert to clinical use.

## Conclusions

The spatiotemporal pattern of Ezrin/Periaxin expression was involved in the control of myoblast differentiation/fusion, myotube length and size, and myofiber specialization, which was related to the activated PKA-NFAT-MEF2C signaling pathway, providing a novel L-Periaxin/Ezrin joint strategy for the treatment of muscle atrophy, especially in CMT4F (Fig. 9).

## Supplementary Information

**Additional file 1: Table S1.** The sequences of primers of qPCR. **Figure S1.** A peroneal nerve injury (PNI) model was successfully established. **Figure S2.** Transfection efficiency of Ezrin overexpression or knockdown in myoblast cells. **Figure S3.** Transfection efficiency of L-Periaxin overexpression or knockdown into C2C12 myoblast cells. **Figure S4.** Low levels of L-Periaxin were required for Ezrin to activate MyoG/MEF2C-mediated myoblast differentiation/fusion. **Figure S5.** The traits of L-Periaxin expression during myoblast differentiation. **Figure S6.** Ezrin regulated myoblast differentiation and myofiber specialization. **Figure S7.** Ezrin regulated myoblast differentiation and fusion through the PKA signaling pathway. **Figure S8.** Ezrin regulated myoblast differentiation and fusion through the PKA signaling pathway. **Figure S9.** NFAT nuclear translocation in Ezrin-mediated myoblast differentiation/fusion. **Figure S10.** NFATs involved in Ezrin-mediated myoblast differentiation/fusion. **Figure S11.** Ezrin regulated myoblast differentiation and fusion through the NFAT-MyoD/MEF2C signaling pathway.

## Data Availability

Please contact the corresponding author for data requests.

## Abbreviations

Ad: Adenovirus
CMT: Charcot-Marie-Tooth disease
CMT4F: Charcot-Marie-Tooth disease type 4F
DAPI: 4´,6-Diamidino-2-phenylindole
DMEM: Dulbecco’s modified Eagle’s medium
DM: Differentiation medium
FBS: Fetal bovine serum
HF: Heart failure
HRP: Horseradish peroxidase
HS: Horse serum
IF: Immunofluorescence
MyHC: Myosin heavy chain
NFAT: Nuclear factor of activated T cells
MEF2C: Myocyte-specific enhancer factor 2C
MyoD: Myogenic differentiation 1
M: Mol/L
PKA: Protein kinase A
PKA-γ cat: Protein kinase A gamma catalytic subunit
PKA R Iα: Protein kinase A alpha regulatory subunit
PM: Proliferation medium
PVDF: Polyvinylidene fluoride
shRNA: Short hairpin RNA
