# Supplementary material for: The spatiotemporal matching pattern of Ezrin/Periaxin involved in myoblast differentiation and fusion and Charcot-Marie-Tooth disease-associated muscle atrophy
Source: J Transl Med. 2023 Mar 4;21:173. doi: 10.1186/s12967-023-04016-7 (PMC9985213; doi:10.1186/s12967-023-04016-7)
Supplement: Supplementary file 1 — Additional file 1: Table S1. The sequences of primers of qPCR. Figure S1. A peroneal nerve injury (PNI) model was successfully established. Figure S2. Transfection efficiency of Ezrin overexpression or knockdown in myoblast cells. Figure S3. Transfection efficiency of L-Periaxin overexpression or knockdown into C2C12 myoblast cells. Figure S4. Low levels of L-Periaxin were required for Ezrin to activate MyoG/MEF2C-mediated myoblast differentiation/fusion. Figure S5. The traits of L-Periaxin expression during myoblast differentiation. Figure S6. Ezrin regulated myoblast differentiation and myofiber specialization. Figure S7. Ezrin regulated myoblast differentiation and fusion through the PKA signaling pathway. Figure S8. Ezrin regulated myoblast differentiation and fusion through the PKA signaling pathway. Figure S9. NFAT nuclear translocation in Ezrin-mediated myoblast differentiation/fusion. Figure S10. NFATs involved in Ezrin-mediated myoblast differentiation/fusion. Figure S11. Ezrin regulated myoblast differentiation and fusion through the NFAT-MyoD/MEF2C signaling pathway. [file 12967_2023_4016_MOESM1_ESM.docx]

**The spatiotemporal matching pattern of Ezrin/Periaxin involved in myoblast differentiation and fusion and Charcot-Marie-Tooth disease-associated muscle atrophy**

Ruo-nan Zhang^1,3,4#^, Xin Bao^1,3,6#^, Yun Liu^3#^, Yan Wang^3#^, Xing-Yuan Li^3,5#^, Ge Tan^3#^, Magdaleena Naemi Mbadhi^3^, Wei Xu^3^, Qian Yang^2,3^, Lu-yuan Yao^2,3^, Long Chen^6^, Xiao-ying Zhao^3^, Chang-qing Hu^3^, Jing-xuan Zhang^3^, Hong-tao Zheng^3^, Yan Wu^3^, Shan Li^7^, Shao-juan Chen^8^, Shi-you Chen^9^, Jing Lv^2*^, Liu-liu Shi^3*^, Jun-ming Tang^1,2,3*^

^1^Faculty of Basic Medical Sciences, Postgraduate Union Training Basement of Jin Zhou Medical University, Hubei University of Medicine, Shiyan, Hubei 442000, PR China

^2^Institute of Anesthesiology, Department of Anesthesiology, Taihe Hospital, Hubei University of Medicine, Shiyan, Hubei 442000, PR China

^3^Department of Physiology, Hubei Key Laboratory of Embryonic Stem Cell Research, Faculty of Basic Medical Sciences, Hubei University of Medicine, Shiyan, Hubei 442000, PR China.

^4^Emergency Comprehensive Department, Shiyan Maternal and Child Health Hospital, Hubei University of Medicine, Shiyan, Hubei 442000, PR China.

^5^Department of Physiology, Faculty of Basic Medical Sciences, Zunyi Medical University, Zunyi, Guizhou 563006, PR China.

^6^Experimental Medical Center, Dongfeng Hospital, Hubei University of Medicine, Shiyan, China.

^7^Department of Biochemistry, Faculty of Basic Medical Sciences, Hubei University of Medicine, Shiyan, 442000, Hubei, People's Republic of China.

^8^Department of Stomatology, Taihe Hospital, Hubei University of Medicine, Shiyan, 442000, Hubei, People's Republic of China.

^9^Department of Surgery, University of Missouri, Columbia, USA.

^#^Co-first author

*Corresponding Author: Jing Lv, Liu-liu Shi & Jun-ming Tang

Department of Physiology, Hubei Key Laboratory of Embryonic Stem Cell Research, Faculty of Basic Medical Sciences, Hubei University of Medicine, Shiyan, Hubei 442000, PR China

Phone: 86-0719-8875312

Email: tangjm416@163.com (Tang); shi-liuliu@163.com (Shi); [389514970@qq.com(Lv)](mailto:389514970@qq.com(Lv))

**Supplemental Table1: The sequences of primers of qPCR.**

| **Gene** | **Forward** | **Reverse** |
| --- | --- | --- |
| MyoG | 5’-GAGACATCCCCCTATTTCTACCA-3’ | 5’-GCTCAGTCCGCTCATAGCC-3’ |
| MyoD1 | 5’-CCACTCCGGGACATAGACTTG-3’ | 5’-AAAAGCGCAGGTCTGGTGAG-3’ |
| MyHC1 | 5’-CAAGCAGCAGTTGGATGAGCGACT-3’ | 5’-TCCTCCAGCTCCTCGATGCGT-3’ |
| MyHC2a | 5’-AGAGGACGACTGCAGACCGAAT-3’ | 5’-GAGTGAATGCTTGCTTCCCCCTTG-3’ |
| MyHC2b | 5’-ACGCTTGCACACAGAGTCAG-3’ | 5’-CTTGGACTCTTCCTCTAGCTGCC-3’ |
| MyHC2x | 5’-ACCAAGGAGGAGGAACAGCAGC-3’ | 5’-GAATGCCTGTTTGCCCCTGGAG-3’ |
| GAPDH | 5’-ATGACTCCACTCACGGCAAA-3’ | 5’-ATGATGACCCTTTTGGCTCC-3’ |

qPCRs were performed to identified satellite cell differentiation and muscle fibers traits by using the specific primers of satellite cell differentiation markers including MyoD and MyoG, type I muscle fiber makers like MyHC1, and type II muscle fiber makers such as MyHC2a, MyHC2b, and MyHC2X.

**Supplemental Figure**

**Figure S1. A peroneal nerve injury (PNI) model was successfully established**


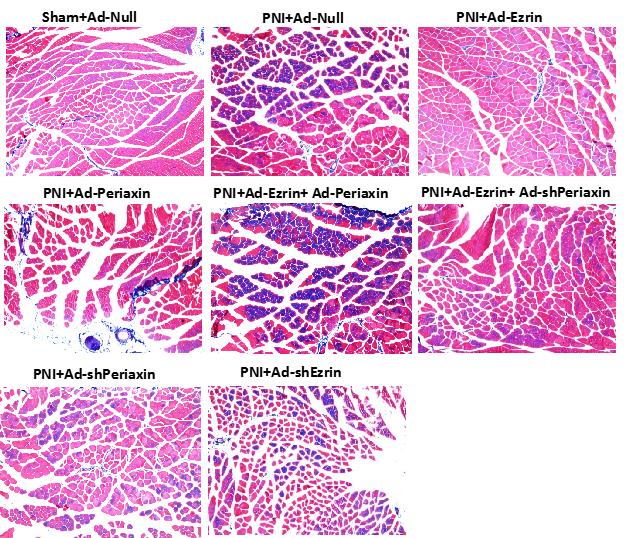


**Figure S1. A peroneal nerve injury (PNI) model was successfully established.**

To further confirm whether Ezrin and L-Periaxin are involved in myoblast differentiation and myofiber specialization, we established a peroneal nerve injury (PNI) model to partially mimic CMT-associated muscle atrophy, and the results showed the traits of gastrocnemius muscle atrophy. Meanwhile, we found that overexpression of Ezrin markedly reduced muscle fibrosis. Masson staining for gastrocnemius muscle treated with Ad-Ezrin or Ad-shEzrin with or without Ad-Periaxi or Ad-shPeriaxin in a peroneal nerve injury (PNI) model.

**Figure S2. Transfection efficiency of Ezrin overexpression or knockdown in myoblast cells**

**
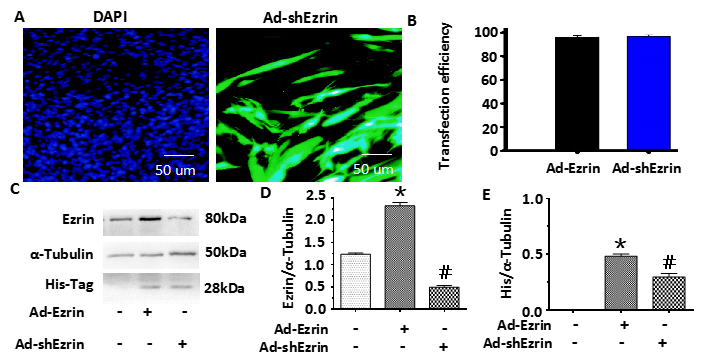
**

**Figure S2. Transfection efficiency of Ezrin overexpression or knockdown in myoblast cells**

We found that 48 hours after transfection with Ad-Ezrin and Ad-shEzrin, their transfection efficiency was more than 90%, suggesting that the transfection was successful. Moreover, transfection of Ad-Ezrin significantly increased Ezrin levels by 150%, while Ad-shEzrin was significantly lower than the control level by 70%. (A) Typical image of adenovirus-mediated overexpression of Ezrin and knockdown of Ezrin by shRNA in myoblast cells transfected with 100 for optimal multiplication of infection (MOI). (B) Quantitative analysis of the transfection efficiency of this specific adenovirus into C2C12 myoblast cells reached almost 95%. (C) Western blot for Ezrin or his-tag expression in myoblast cells treated by overexpression or knockdown of Ezrin for 6 days. (D-E) A quantitative assay for Ezrin or His-tag expression was performed 6 days after myoblast differentiation. n=3, **P<0.05* vs. Ctrl.


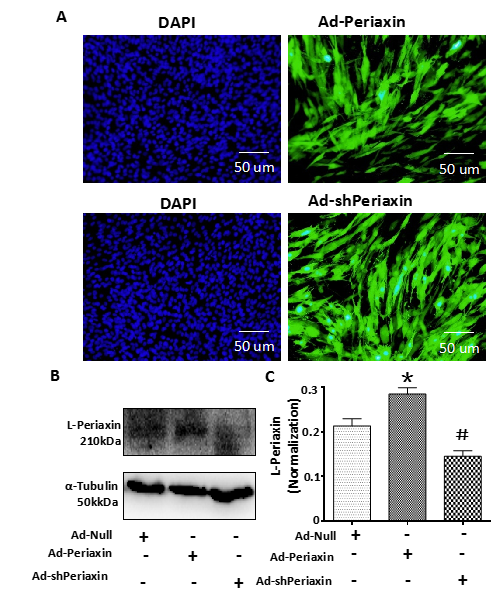


**Figure S3. Transfection efficiency of L-Periaxin overexpression or knockdown into C2C12 myoblast cells**

We found that 48 hours after transfection of Ad-L-Periaxin and Ad-shL-Periaxin, their transfection efficiency was more than 90%, suggesting that the transfection was successful. Moreover, overexpression transfection significantly increased a by 50%, while knockdown treatment was significantly lower than the control level by 50%. (A) Typical image of adenovirus-mediated overexpression of L-Periaxin and knockdown of L-Periaxin by shRNA in myoblast cells transfected with 100 optimal multiplication of infection (MOI). Quantitative analysis of the transfection efficiency of this specific adenovirus into C2C12 myoblast cells reached almost 97%. (B) Western blot for L-Periaxin expression in myoblast cells treated by overexpression or knockdown of L-Periaxin for 6 days. (C) Quantitative assay for L-Periaxin expression was performed 6 days after myoblast differentiation. n=3, **P<0.05* vs.Ad-Null; *^#^P<0.05* vs. Ad-Null.

**Figure S4. Low levels of L-Periaxin were required for Ezrin to activate MyoG/MEF2C-mediated myoblast differentiation/fusion**


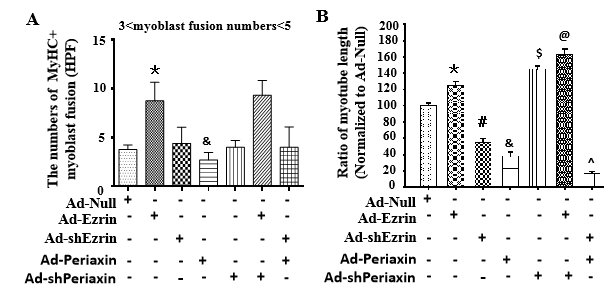


**Figure S4. Low levels of L-Periaxin were required for Ezrin to activate MyoG/MEF2C-mediated myoblast differentiation/fusion**

1. Ad-Ezrin increased the numbers of MyHC+ myotubes with more than 3 and less than 5 myoblast fusions, while Ad-Periaxin decreased them, as determined by quantitative assays of myotubes. The specific effects could not be abolished by Ad-shPeriaxin or Ad-shezin, respectively. (B) Ad-Ezrin or Ad-shPeriaxin increased myotube length in MyHC-positive myotubes with 5^+^ myoblast fusion, while Ad-shEzrin or Ad-Periaxin decreased them, as analyzed by quantitative assays of the ratio of myotube length normalized to the Ad-null group. Ad-shPeriaxin enhanced the role of Ad-Ezrin in myotube size, while Ad-Periaxin further deteriorated the inhibitory role of Ad-shEzrin in myotube length. Three independent experiments were performed, n=3, **P<0.05* vs. Ad-Null; *^#^P<0.05* vs. Ad-Null; *^&^P<0.05* vs. Ad-Null; *^$^P<0.05* vs. Ad-Null; *^@^P<0.05* vs. Ad-Ezrin*; ^P<0.05* vs. Ad-shEzrin.

**Figure S5. The traits of L-Periaxin expression during myoblast differentiation**


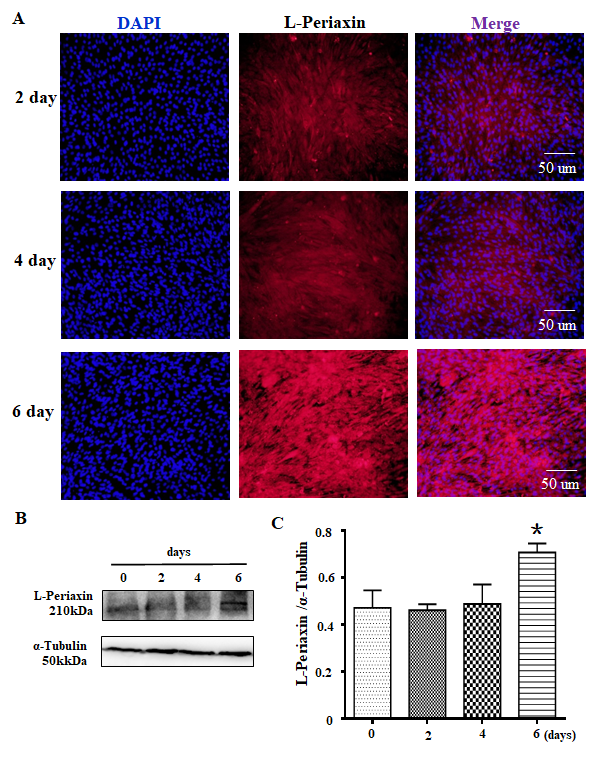


**Figure S5. The traits of L-Periaxin expression during myoblast differentiation**

We found that L-Periaxin expression gradually increased and peaked at 6 days of myoblast differentiation, indicating that L-Periaxin could affect myoblast differentiation. (A) Typical image of L-Periaxin staining in differentiated C2C12 myoblasts treated with Ezrin overexpression or knockdown for 2, 4 and 6 days. (B-C) Quantitative assays for L-Periaxin were performed 2, 4 and 6 days after myoblast differentiation. n=3, **P<0.05* vs. 0, 2 and 4 days.

**Figure S6. Ezrin regulated myoblast differentiation and myofiber specialization**


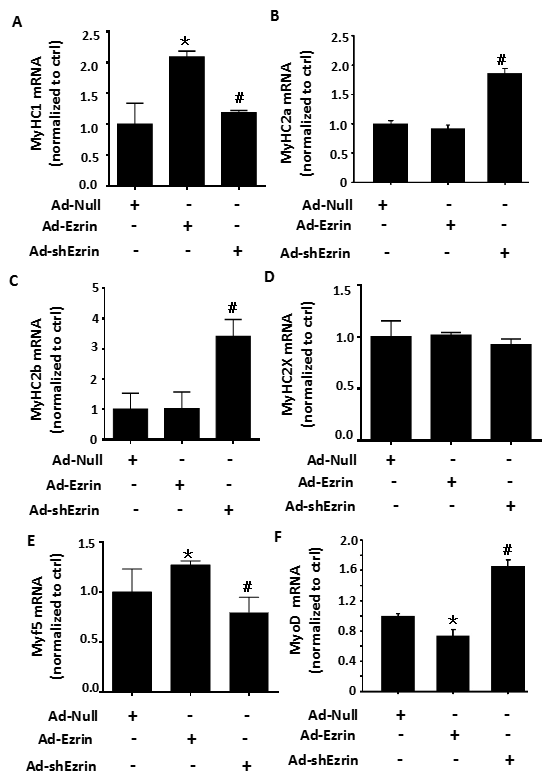


**Figure S6. Ezrin regulated myoblast differentiation and myofiber specialization**

We found that overexpressing Ezrin obviously increased ‘slow muscle’ MyHC-1 mRNA levels. Conversely, knockdown of Ezrin by shRNA slightly increased MyHC-2a and MyHC-2b mRNA levels. (A-D) Overexpression of Ezrin promoted MyHC-1 expression, while knockdown of Ezrin increased the levels of type II muscle fiber markers, such as MyHC-2a and MyHC-2b, in differentiated C2C12 cells pretreated with Ad-AdEzrin or Ad-shEzrin, as shown by detecting the mRNA levels using real-time PCR. (E-F) Ad-Ezrin increased Myf5 mRNA levels while decreasing MyoD mRNA levels, as determined by real-time PCR. Conversely, Ad-shEzrin increased MyoD mRNA levels while decreasing Myf5 mRNA levels. n=3, **P<0.05* vs. Ad-Null; *^#^P<0.05* vs. Ad-Null.

**Figure S7. Ezrin regulated myoblast differentiation and fusion through the PKA signaling pathway**

**
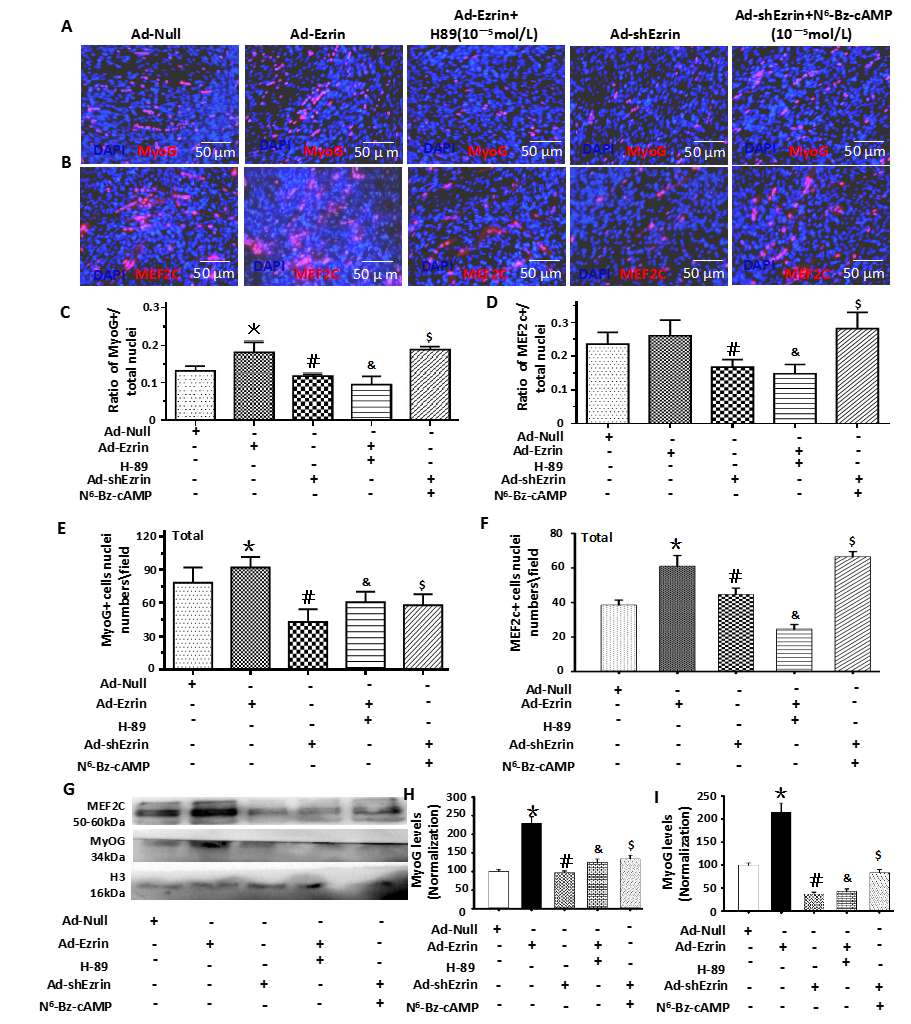
**

**Figure S7. Ezrin regulated myoblast differentiation and fusion through the PKA signaling pathway**

We found that knockdown of Ezrin by shRNA markedly decreased MyoG^+^ and MEF2C^+^ in fewer than 3 nuclei, and these effects could be abolished by a PKA activator. In contrast, overexpression of Ezrin substantially increased the number of MyoG+ or MEF2C+ nuclei in fewer than 3 and 3+ myotubes, and these effects could be abolished by the PKA inhibitor H-89. Furthermore, western blot analysis showed that overexpression of Ezrin increased the nuclear levels of MyoG and MEF2C, while knockdown of Ezrin by shRNA reduced these levels. More importantly, these specific changes could be cancelled by the PKA inhibitor and PKA activator. These results indicated that Ezrin participated in C2C12 myoblast differentiation and fusion through the PKA-MyoG/MEF2C signaling pathway. (A-B) Typical image of MyoG and MEF2c staining in differentiated C2C12 myoblasts treated with Ad-Ezrin with or without H-89 (10^-^5 mol/L) or Ad-shEzrin with or without N^6^-Bz-cAMP (10^-^5 mol/L), respectively. (C-F) Quantitative assay for the number of MyoG+ or MEF2c+ nuclei was analyzed 6 days after myoblast differentiation treated with Ad-Ezrin with or without H-89 or Ad-shEzrin with or without N^6^-Bz-cAMP (10^-^5 mol/L). n=3, **P<0.05* vs. Ctrl; *#P<0.05* vs. Ad-*Ezrin; &P<0.05* vs. Ctrl; *$P<0.05* vs. Ad-shEzrin. (G) Western blot for the indicated proteins in myoblasts treated with Ezrin overexpression or knockdown for 6 days. (H-I) Quantitative assays for the indicated proteins were performed 6 days after myoblast differentiation following Ezrin overexpression or knockdown. n=3, **P<0.05* vs. Ad-Null; *^#^P<0.05* vs. Ad-Ezrin*; ^&^P<0.05* vs. Ad-Null; *^$^P<0.05* vs. Ad-shEzrin.

**Figure S8. Ezrin regulated myoblast differentiation and fusion through the PKA signaling pathway**


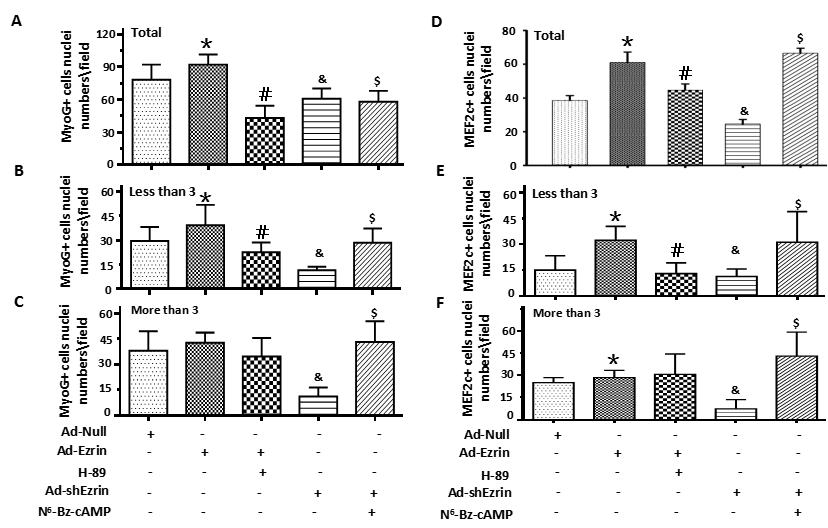


**Figure S8. Ezrin regulated myoblast differentiation and fusion through the PKA signaling pathway**

We found that knockdown of Ezrin by shRNA markedly decreased MyoG^+^ and MEF2C^+^ in fewer than 3 nuclei, and these effects could be abolished by a PKA activator. In contrast, overexpression of Ezrin substantially increased the number of MyoG+ or MEF2C+ nuclei in fewer than 3 and 3+ myotubes, and these effects could be abolished by the PKA inhibitor H-89. These results indicated that Ezrin participated in C2C12 myoblast differentiation and fusion through the PKA-MyoG/MEF2C signaling pathway. (A-F) Quantitative assay for the number of MyoG+ or MEF2c+ nuclei in sFigure 6 was analyzed 6 days after myoblast differentiation treated with Ad-Ezrin with or without H-89 or Ad-shEzrin with or without N^6^-Bz-cAMP (10^-^5 mol/L. n=3, **P<0.05* vs. Ad-Null; *^#^P<0.05* vs. Ad-Ezrin*; ^&^P<0.05* vs. Ad-Null; *^$^P<0.05* vs. Ad-shEzrin.

**Figure S9. NFAT** **nuclear translocation in Ezrin-mediated myoblast differentiation/fusion**

**
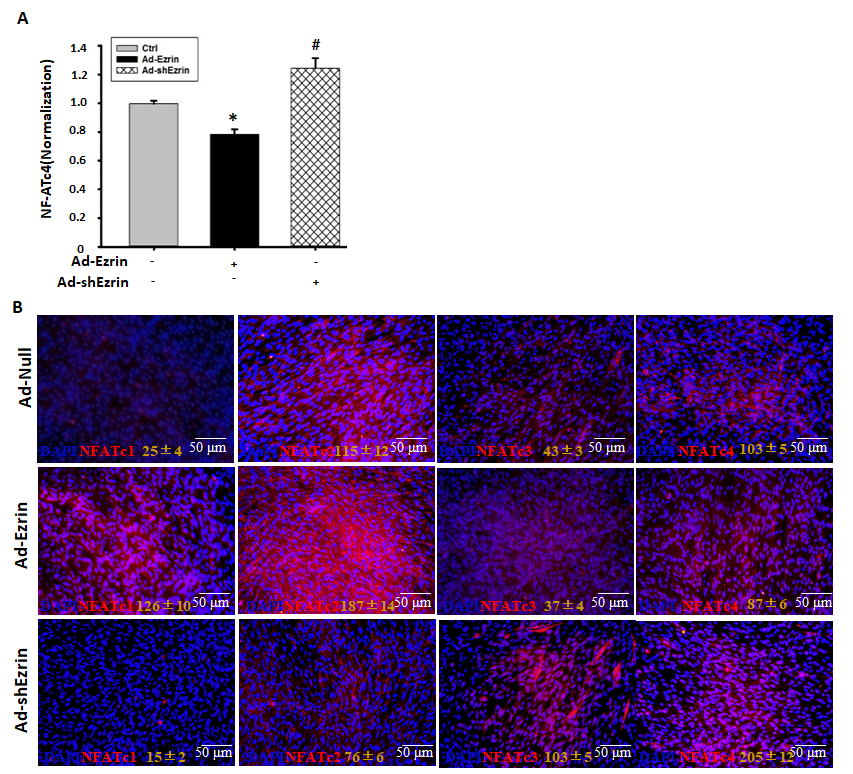
**

**Figure S9. NFAT** **nuclear translocation in Ezrin-mediated myoblast differentiation/fusion**

We found that the overexpression of Ezrin promoted the nuclear translocation of NFATc1/c2, which led to increased levels of NFATc1/c2 nuclei and decreased NFATc3/c4 nuclei levels. Conversely, knocking down Ezrin induced nuclear translocation of NFATc3/c4, resulting in increased NFATc3/c4 nuclear levels and decreased NFATc1/c2 nuclear levels. (A) A quantitative assay of NFATc4 proteins was performed 6 days after myoblast differentiation following Ezrin overexpression or knockdown. Three independent experiments were performed, n=3, **P<0.05* vs. Ad-Null (Ctrl); *^#^P<0.05* vs. Ad-Null (Ctrl). (B) Typical image and semiquantitative assay of NFATc1-c4 nuclear translocation at 6 days after myoblast differentiation following Ezrin overexpression or knockdown. Red fluorescence indicates NFATc1-c4; DAPI indicates the nucleus.

**Figure S10. NFATs involved in Ezrin-mediated myoblast differentiation/fusion**


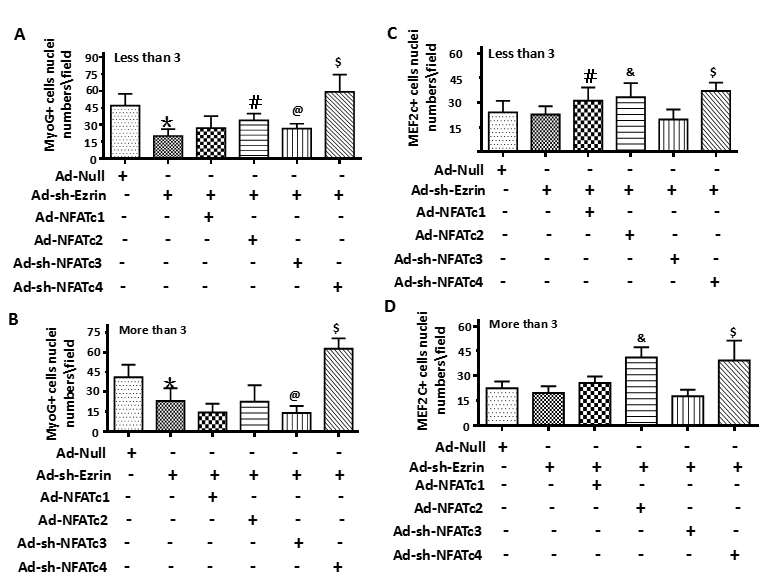


**Figure S10. NFATs involved in Ezrin-mediated myoblast differentiation/fusion**

We found that knockdown of Ezrin by shRNA markedly decreased the number and percentage of MyoG+ and MEF2C+ nuclei in less than 3-nuclei cells and 3-nuclei^+^myotubes, and treatment with Ad-NFATc2 or Ad-shNFATc4 abolished these effects. In addition, Ad-NFATc1 or Ad-shNFATc3 reversed the number of MEF2C+ nuclei in 3-nuclei^+^myotubes. These results indicated that Ezrin participated in C2C12 myoblast differentiation and fusion with the coordination of MyoG and MEF2C, which were associated with NFATc2/c4, at least in part. (A-D) Quantitative assay for the number of MyoG+ or MEF2c+ nuclei was analyzed 6 days after myoblast differentiation treated with Ad-shEzrin with or without Ad-NFATc1, Ad-NFATc2, Ad-shNFATc3 and Ad-shNFATc4. n=3, **P<0.05* vs. Ad-Null; *#P<0.05* vs. Ad-shEzrin; *^&^P<0.05* vs. Ad-shEzrin; *^$^P<0.05* vs. Ad-shEzrin.

**
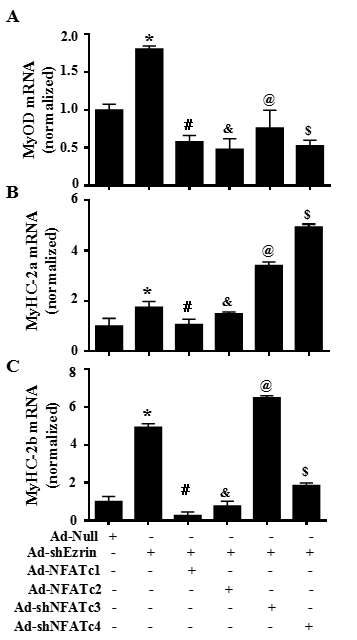
**

**Figure S11. Ezrin regulated myoblast differentiation and fusion through the NFAT-MyoD/MEF2C signaling pathway**

We found that overexpression of Ezrin increased the numbers of MyHC-I- and MyHC-II-positive myotubes, while knockdown of Ezrin decreased these numbers, and the specific effects could be abolished by Ad-NFATc1/c2 or Ad-shNFATc3/c4. More importantly, the specific increase in MyHC-2a and MyHC-2b mediated by Ezrin knockdown was obviously reversed by Ad-NFATc1/c2 or Ad-shNFATc4, respectively. Thus, Ezrin mainly regulated myofiber specification through the integrated role of the NFAT signaling pathway. (A-C) Real-time PCR for MyoD, MyHC-2a and MyHC-2b in Ezrin-knockdown myobalst cells pretreated with Ad-NFATc1/c2 or Ad-shNFATc3/c4. n=3, **P<0.05* vs. Ad-Null; *^#^P<0.05* vs. Ad-shEzrin; *^&^P<0.05* vs. Ad-shEzrin; *^$^P<0.05* vs. Ad-shEzrin; *^@^P<0.05* vs. Ad-shEzrin.
